# Covalent Polymer‐RNA Conjugates for Potent Activation of the RIG‐I Pathway

**DOI:** 10.1002/adhm.202303815

**Published:** 2024-05-03

**Authors:** Christian R. Palmer, Lucinda E. Pastora, Blaise R. Kimmel, Hayden M. Pagendarm, Alexander J. Kwiatkowski, Payton T. Stone, Karan Arora, Nora Francini, Olga Fedorova, Anna M. Pyle, John T. Wilson

**Affiliations:** ^1^ Department of Chemical and Biomolecular Engineering Vanderbilt University Nashville TN 37235 USA; ^2^ Department of Biomedical Engineering Vanderbilt University Nashville TN 37235 USA; ^3^ Department of Molecular Cellular and Developmental Biology Yale University New Haven CT 06511 USA; ^4^ Howard Hughes Medical Institute Chevy Chase MD 20815 USA; ^5^ Department of Chemistry Yale University New Haven CT 06511 USA; ^6^ Department of Pathology Microbiology and Immunology Vanderbilt University Medical Center Nashville TN 37232 USA; ^7^ Vanderbilt‐Ingram Cancer Center Vanderbilt University Medical Center Nashville TN 37232 USA

**Keywords:** bioconjugation, innate immunity, nanoparticle, nucleic acid therapeutic, oligonucleotide, polymer, RIG‐I

## Abstract

RNA ligands of retinoic acid‐inducible gene I (RIG‐I) are a promising class of oligonucleotide therapeutics with broad potential as antiviral agents, vaccine adjuvants, and cancer immunotherapies. However, their translation has been limited by major drug delivery barriers, including poor cellular uptake, nuclease degradation, and an inability to access the cytosol where RIG‐I is localized. Here this challenge is addressed by engineering nanoparticles that harness covalent conjugation of 5′‐triphospate RNA (3pRNA) to endosome‐destabilizing polymers. Compared to 3pRNA loaded into analogous nanoparticles via electrostatic interactions, it is found that covalent conjugation of 3pRNA improves loading efficiency, enhances immunostimulatory activity, protects against nuclease degradation, and improves serum stability. Additionally, it is found that 3pRNA could be conjugated via either a disulfide or thioether linkage, but that the latter is only permissible if conjugated distal to the 5′‐triphosphate group. Finally, administration of 3pRNA‐polymer conjugates to mice significantly increases type‐I interferon levels relative to analogous carriers that use electrostatic 3pRNA loading. Collectively, these studies have yielded a next‐generation polymeric carrier for in vivo delivery of 3pRNA, while also elucidating new chemical design principles for covalent conjugation of 3pRNA with potential to inform the further development of therapeutics and delivery technologies for pharmacological activation of RIG‐I.

## Graphical abstract

Polymeric carriers are engineered for covalent conjugation of oligonucleotide agonists of retinoic acid‐inducible gene I (RIG‐I) and chemical design criteria for optimizing their immunostimulatory activity are established.

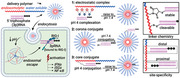

## 1 Introduction

Retinoic acid‐inducible gene I (RIG‐I) is a cytosolic pattern recognition receptor (PRR) that potently mediates antiviral innate immunity upon binding of blunt‐ended 5′ di‐ or triphosphorylated double‐stranded RNA (2p‐ or 3pRNA).^[^ ^1^, ^2^, ^3^ ^]^ Activation of RIG‐I triggers an innate immune response characterized by the expression of type‐I interferons (IFN‐I), IFN‐stimulated genes (ISGs), and Th1 cytokines that potentiate diverse antiviral effector functions.^[^ ^4^, ^5^, ^6^ ^]^ Accordingly, 3pRNA has been explored for pharmacological inhibition of viral infection in models of influenza and SARS‐CoV‐2,^[^ ^7^, ^8^, ^9^ ^]^ and as a vaccine adjuvant in a variety of disease models.^[^ ^10^, ^11^ ^]^ Additionally, RIG‐I expression has been shown to correlate with tumor T‐cell infiltration, survival, and overall outcomes in melanoma patients treated with anti‐CTLA‐4 immunotherapy, motivating the exploration of RIG‐I agonists in immuno‐oncology.^[^ ^12^, ^13^ ^]^ When packaged with polycationic transfection agents, intratumoral administration of 3pRNA can initiate RIG‐I‐mediated tumor inflammation and improve response to immune checkpoint inhibitors in multiple murine tumor models.^[^ ^14^, ^15^ ^]^ Based on these findings, intralesional delivery of RIG‐I agonists has advanced into clinical trials (e.g., NCT03065023, NCT03739138).^[^ ^16^ ^]^

Despite high clinical potential, 3pRNA faces multiple and substantial drug delivery barriers that limit its efficacy and utility, including poor cellular uptake, susceptibility to nuclease degradation, and inability to access the cytosol, where RIG‐I is localized.^[^ ^17^, ^18^, ^19^ ^]^ Hence, administration of free 3pRNA does not activate RIG‐I,^[^ ^18^, ^19^, ^20^ ^]^ necessitating its formulation with commercially available materials (e.g., lipofectamine, polyethylenimine) that have not been optimized for 3pRNA.^[^ ^15^, ^21^, ^22^ ^]^ Yet, investigations focused on optimizing carriers for 3pRNA are relatively few,^[^ ^15^, ^21^, ^22^, ^23^ ^]^ particularly when compared to other nucleic acid therapeutics, including siRNA, miRNA, DNA, and mRNA.^[^ ^24^, ^25^, ^26^, ^27^, ^28^, ^29^ ^]^ While 3pRNA shares delivery barriers with other classes of nucleic acids, it is also distinguished by its structure and immunopharmacological mechanism of action. This motivates the design of carriers that are specifically optimized for 3pRNA cargo.

Toward closing this technology gap, our group has recently investigated structure‐function‐activity relationships using a library of 30 rationally designed poly[(ethylene glycol monomethyl ether) (mPEG)‐*b*‐(dimethylaminoethyl methacrylate (DMAEMA)‐*c*‐alkyl methacrylate)] (mPEG‐*b*‐DA) diblock copolymers as carriers for 3pRNA.^[^ ^23^ ^]^ The cationic DMAEMA and hydrophobic alkyl methacrylate groups in the DA blocks facilitated electrostatic complexation with 3pRNA into nanoparticles (NPs) and imparted pH‐responsive, endosomolytic behavior, while the mPEG block served to shield the complex and imparted surface charge‐neutrality, water‐solubility, and colloidal stability. Four potential lead carriers for 3pRNA were identified based on their capacity to facilitate endosomal escape, stimulate RIG‐I‐dependent inflammatory responses in vitro, and activate RIG‐I signaling in healthy mice following intravenous administration. While this represented an important technological advancement toward the development of carriers for harnessing the potential of RIG‐I agonists, we nonetheless identified several important limitations in this approach. First, electrostatic loading of 3pRNA with mPEG‐*b*‐DA polymers provided incomplete protection from serum destabilization of NPs, a common limitation of polycationic nucleic acid carriers that is a consequence of decomplexation by serum proteins and/or poor protection from RNases.^[^ ^30^, ^31^ ^]^ Additionally, relatively high polymer:3pRNA ratios were required in order to adequately encapsulate the 3pRNA cargo. Because NPs (particularly those with cationic elements) can cause carrier‐mediated, off‐target toxicities,^[^ ^32^ ^]^ it is important to reduce the amount of polymer required to encapsulate, protect, and efficiently deliver 3pRNA.

**Figure 1:**
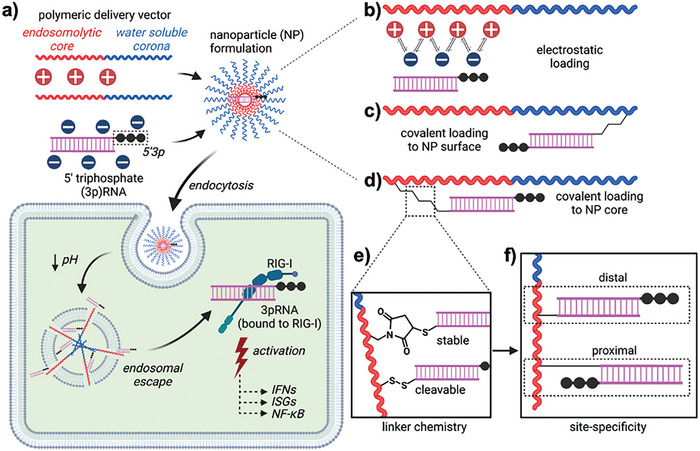
Design concepts for polymer nanocarriers of 3pRNA investigated in this study. a) Polymeric nanoparticles (NPs) are capable of loading 3pRNA and promoting endosomal escape to facilitate delivery to the cytosol, resulting in activation of RIG‐I signaling. b–d) Illustrations depicting the loading strategies employed by b) polymer 1 (electrostatic complexation to core of NP), c) polymer 2 (conjugation of 3pRNA to NP corona), and d) polymer 3 (conjugation of 3pRNA to the core‐forming block of the NP). e) Cutout contrasting the two covalent attachment chemistries investigated herein. f) Illustration highlighting the two opposing orientations of polymer‐3pRNA conjugates (distal and proximal) examined in this work. Created using BioRender.com.

We postulated that covalent conjugation of 3pRNA to the polymer backbone could increase the efficiency of RNA loading while affording enhanced stability and protection from serum‐mediated degradation (**Figure** **1**). Indeed, previous efforts have leveraged covalent ligation of other classes of oligonucleotide therapeutics (primarily siRNA) to diverse carriers, including NPs, lipids, peptides, and others.^[^ ^33^, ^34^, ^35^, ^36^, ^37^, ^38^, ^39^, ^40^, ^41^, ^42^ ^]^ However, we are unaware of any efforts to covalently conjugate 3pRNA to polymers or nanoparticles with the exception of our previous work, in which we synthesized 3pRNA‐mPEG conjugates to delineate the effects of molecular weight (MW), linker cleavability, and conjugation site on the immunostimulatory activity of 3pRNA.^[^ ^43^ ^]^ These studies provided insight into the chemical design rules for the synthesis of 3pRNA conjugates and established a foundation for leveraging covalent conjugation as a strategy for improving 3pRNA delivery using our DA‐based polymer carriers.

We selected a lead carrier for 3pRNA delivery from our previous work, in which the DA block was composed of 50% DMAEMA and 50% *n*‐butyl methacrylate (BMA) (4‐50), and expanded upon this architecture by synthesizing polymers with a poly[(DMAEMA‐*c*‐BMA) (DB)‐*block*‐(dimethyl acrylamide) (DMA)] structure (Figure 1).^[^ ^23^, ^44^ ^]^ As described above, the 4–50 block facilitates electrostatic interactions with 3pRNA and promotes endosomal escape, while the polyDMA block substitutes for mPEG to provide the desired water solubility and charge‐neutral shielding criteria (Figure 1a,b). Critically, we replaced the mPEG block with polyDMA to enable copolymerization of thiol‐reactive pyridyl disulfide (PDS)‐functionalized monomers in the corona‐forming second block for covalent conjugation of thiol‐modified 3pRNA (Figure 1c). By also synthesizing structural analogs with pendant PDS groups instead copolymerized in the 4–50 block (Figure 1d), this design also allows the delineation of the effect of “corona” versus “core” conjugation of thiol‐modified 3pRNA, a question of fundamental interest for 3pRNA carrier design. Employing this strategy, we investigated how covalent conjugation of 3pRNA to polymer carrier – either to the corona‐ or core‐forming segments – affects the physiochemical and biological properties and activities of 3pRNA‐loaded NPs compared to those assembled only via electrostatic complexation. Additionally, we assessed the impact of cleavable disulfide versus stable thioether conjugate linker chemistry (Figure 1e). Further, we evaluated the impact of conjugating 3pRNA via thiol modifiers installed distal or proximal to the 5′‐triphosphate group (i.e., on the 5′ or 3′ end of the complement strand, respectively) through these two attachment chemistries to interrogate the role of 3pRNA orientation (Figure 1f). By exploring this parameter space, we have established new chemical design principles for the packaging and delivery of 3pRNA and have leveraged these principles to engineer a next‐generation nanocarrier platform that harnesses covalent conjugation of 3pRNA for potent activation of RIG‐I.

## 2 Results and Discussion

### 2.1 Synthesis and Characterization of Endosomolytic Polymers for Delivery of 3pRNA

**Figure 2:**
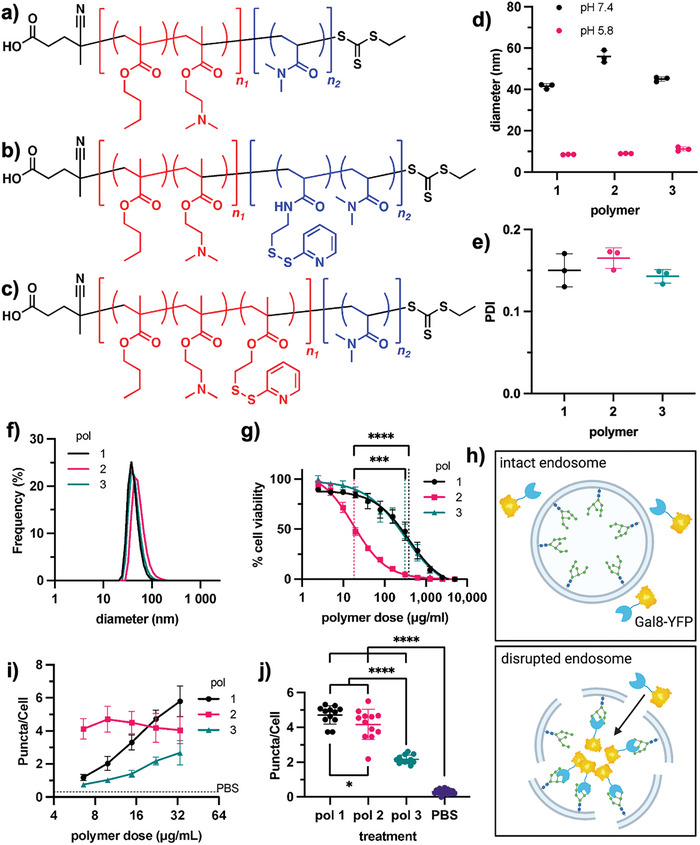
Structure and properties of polymeric nanocarriers for 3pRNA. a–c) Skeletal structures of a) polymer 1, b) polymer 2, and c) polymer 3. d) Z‐Average mean diameters of indicated polymer nanoparticles as measured by dynamic light scattering (DLS) at indicated pH. e) Polydispersity indices (PDIs) of the pH 7.4 particle diameter measurements in d). f) Representative histograms of the DLS measurements at pH 7.4 illustrated in d) and e); pol: polymer. g) Viability of A549 cells treated with indicated polymers and concentrations. Dotted vertical lines depict calculated half‐maximal inhibitory concentrations. Data representative of three independent experiments. h) Illustration depicting the mechanism of the Gal8‐YFP recruitment assay for measuring endosomal escape. Created using BioRender.com. i) Enumeration of fluorescent puncta per cell of Gal8‐YFP engineered MDA‐MB‐231 cells treated with indicated polymers and doses. Data representative of two independent experiments. j) 22.2 µg mL^−1^ data from h), near the IC_50_ of polymer 2. g,j) Comparisons by ordinary one‐way ANOVA with Tukey's multiple comparisons test. d,e) *n* = 3. g) *n* = 4. i,j) *n*_(polymer)_ = 12. *n*_(PBS)_ = 36. All data shown are mean +/− SD. * *P* < 0.05. *** *P* < 0.001. **** *P* < 0.0001.

Reversible addition‐fragmentation chain transfer (RAFT) polymerization was used to synthesize polymers containing a hydrophilic polyDMA block that imparts aqueous solubility following polymerization from a macro‐chain transfer agent (mCTA) comprising a protonatable and hydrophobic 4–50 block to facilitate 3pRNA complexation and NP self‐assembly (**Figure** **2**). Thiol‐reactive PDS‐containing monomers were incorporated at 5 mol% in one of the blocks or withheld from both, enabling the synthesis of polymers for the electrostatic complexation of 3pRNA in addition to those for conjugation of 3pRNA to the “corona” and “core” of the polymer nanocarrier (polymers **1**, **2**, and **3**, respectively, skeleton structures depicted in Figure 2a–c, respectively). PDS groups undergo thiol‐exchange with free thiols to form disulfides that become reduced in the cytosol, allowing for release of 3pRNA.^[^ ^45^, ^46^, ^47^ ^]^ We selected a cleavable disulfide linker chemistry for our initial carrier designs based on our previous work with mPEG‐3pRNA conjugates, in which we showed cleavable linker chemistries are required to maintain the immunostimulatory activity of 3pRNA when 5 kDa mPEG was conjugated to the same end of the 3pRNA as the 5′ triphosphate moiety (i.e., the “proximal” conjugation site, Figure 1f).^[^ ^43^ ^]^ A full summary of polymer properties can be found in Table S1 (Supporting Information). Briefly, the endosomolytic blocks of each polymer had a degree of polymerization (DP) of ≈220, composed of either 0 or ≈10 incorporated PDS‐ethyl methacrylate (PDSEMA) groups for thiol‐mediated conjugation with the balance of the block comprising an equimolar ratio of protonatable DMAEMA and hydrophobic BMA groups (i.e., 4–50). Using these products as mCTAs, a second, hydrophilic block of dimethylacrylamide (DMA) containing 0 or ≈10 incorporated PDS‐ethyl acrylamide (PDSEAm) was chain‐extended from the first blocks to form second blocks with a DP of ≈190. The number‐average MWs (*M*_n_) as estimated by ^1^H‐NMR agree with *M*_n_ estimates from gel permeation chromatography (GPC) measurements for polymers **1** and **2** as well (Figure S1 and Table S1, Supporting Information). This yielded three initial polymers: polymer **1**, containing no conjugable PDS groups and intended to solely form electrostatic complexes, polymer **2**, including PDS groups in the hydrophilic DMA block and intended to form “corona” conjugates of 3pRNA, and polymer **3**, including PDS groups in the endosomolytic 4–50 block and intended to form “core” conjugates of 3pRNA. Dynamic light scattering (DLS) measurements of self‐assembled polymer micelles show consistently sized, low dispersity NPs (PDI < 0.2) at physiological pH (7.4) that transition into smaller species at the late‐endosomal pH of 5.8, indicative of pH‐responsive micelle disassembly driven by protonation of the DMAEMA groups and subsequent solubilization of the second block (Figure 2d,e).^[^ ^23^, ^44^, ^48^, ^49^ ^]^ Representative DLS histograms of polymers **1**–**3** can are shown in Figure 2f.

We next evaluated polymer cytotoxicity in A549 lung carcinoma epithelial cells. Interestingly, we found that polymer **2** exhibited significantly more toxicity than polymers **1** or **3**, resulting in a significantly lower half‐maximal inhibitory concentration (IC_50_ = 18.00 µg mL^−1^, 325 × 10^−9^ m) in comparison to those treated with polymers **1** (IC_50_ = 386.1 µg mL^−1^, 7.35  × 10^−6^ m) and **3** (IC_50_ = 309.0 µg mL^−1^, 5.99  × 10^−6^ m) (Figure 2g). We speculate that this is due to the presence of reactive PDS groups on the NP surface that are capable of reacting with free thiols on cell surface proteins, resulting in increased uptake of polymer.^[^ ^50^ ^]^ To investigate this, we synthesized a variant of polymer **2**, in which the surface PDS groups were reduced to thiols and subsequently capped with *N*‐ethyl maleimide, resulting in a polymer that would not be able to react with cell surface thiols. We then compared the cytotoxicity of polymer **2** with this maleimide‐capped variant and found that replacing the PDS groups resulted in significantly less cytotoxicity, which supports this possible mechanism (Figure S2, Supporting Information). We also employed a Galectin 8 (Gal8) recruitment assay in an engineered MDA‐MB‐231 breast carcinoma cell line to assess the endosomal escape capacity of polymers **1**, **2**, and **3** at a dose near the IC_50_ of polymer **2**. Upon disruption of the endosome, the peptidoglycans of the endosomal lumen are exposed to the cytosolic milieu, including Gal8 fused with yellow fluorescent protein (YFP). Binding of Gal8 to endosomal peptidoglycans results in a redistribution of YFP from diffuse within the cytosol to concentrated as discrete puncta that can be enumerated as a metric of endosomal escape (Figure 2h).^[^ ^51^ ^]^ All three polymers promoted endosomal disruption compared to PBS control as evidenced by an increased number of observed fluorescent puncta (Figure 2i,j). In contrast to polymer **2**, which exhibited endosomolysis at all tested doses, polymers **1** and **3** exhibit dose‐dependent endosomal disruption (Figure 2i). We speculate that the increased activity of polymer **2** may be due to increased cellular internalization mediated by binding to cell surface thiols, which may also contribute to the increased cytotoxicity of polymer **2** as mentioned above. At a dose near the IC_50_ of polymer **2** (22.2 µg mL^−1^ polymer), treatment with polymer **3** results in significantly less endosomal disruption compared to polymers **1** and **2** (Figure 2j) but significantly more than background (PBS vehicle control). This could be due to altered polarity of the pH‐responsive 4–50 block that includes PDS groups, whose constituent aryl disulfide groups are transformed into thiols upon bioreduction. This may reflect the delicate balance of hydrophobicity and composition of incorporated monomers, which we have previously shown can affect pH‐responsive endosomolytic activity.^[^ ^23^ ^]^ Together, these data indicate that these polymers self‐assemble into consistently sized, pH‐responsive nanoparticles that facilitate endosomal escape.

### 2.2 Synthesis and Characterization of 3pRNA/Polymer Complexes and 3pRNA‐Polymer Conjugates

**Figure 3:**
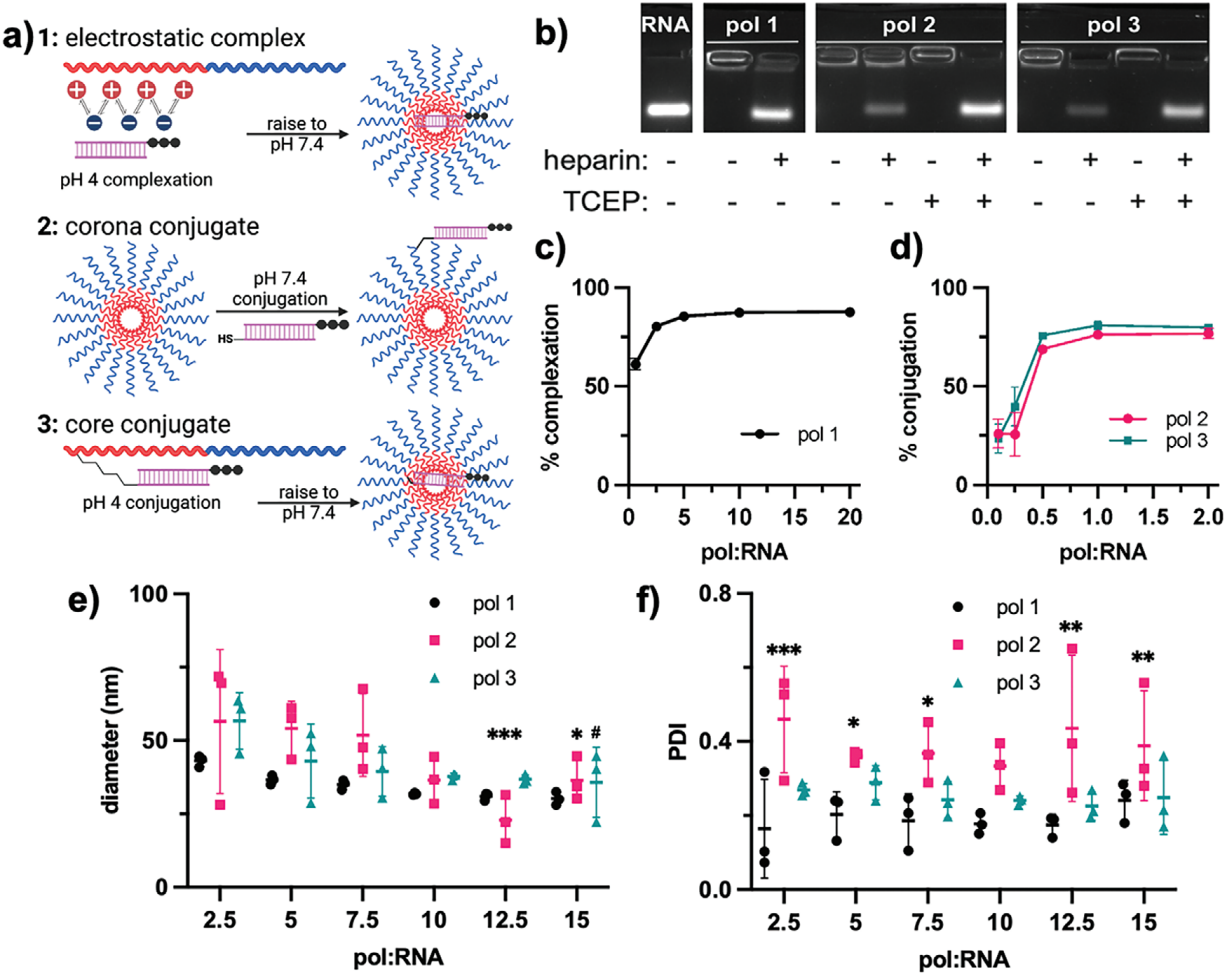
Characterization 3pRNA‐loaded polymer nanoparticles. a) Illustration depicting 3pRNA loading strategies for polymers 1–3, with polymer 1 loading 3pRNA electrostatically and polymers 2 and 3 loading 3pRNA covalently to the corona‐ and core‐forming blocks, respectively. Created using BioRender.com. b) Representative image of electrophoretic mobility shift assay of indicated polymer + 3pRNA formulations (0.50 µg 3pRNA per lane) with and without inclusion of 12.5 units of heparin and/or 10 × 10^−3^ m TCEP as shown; pol: polymer. TCEP: tris(2‐carboxyethyl)phosphine. **c)** Percentage of 3pRNA complexed with polymer **1** as a function of polymer to RNA (pol:RNA) ratio as measured by fluorometric RiboGreen exclusion assay. d) Percentage of 3pRNA conjugated to polymers **2** and **3** at depicted polymer:3pRNA molar ratios as measured by densitometry of heparin^+^ TCEP^−^ bands in Fiji. e) Diameter of 3pRNA/polymer NPs formulated with indicated polymers as measured by dynamic light scattering at depicted polymer:3pRNA molar ratios. Comparisons made by two‐way ANOVA with Dunnett's multiple comparisons test. Comparisons are made between each ratio and pol:3pRNA = 2.5 as a control with each polymer constituting one family. Asterisks (*) indicate comparisons between pol:3pRNA ratios of polymer **2**. Hashes (#) indicate comparisons between pol:3pRNA ratios of polymer **3**. *, ^#^ *P* < 0.05. *** *P* < 0.001. **f)** Polydispersity indices (PDIs) of the measurements in e). Comparisons made by two‐way ANOVA with Dunnett's multiple comparisons test. Comparisons are made between each ratio and empty NPs (data shown in Figure 2e) with each polymer constituting one family. Asterisks (*) indicate comparisons of polymer **2**. * *P* < 0.05. ** *P* < 0.01. *** *P* < 0.001. c–f) *n* = 3 independent formulations. All data shown are mean +/−SD.

The capacity of the polymers to electrostatically complex (polymer **1**), corona conjugate (polymer **2**), and core conjugate (polymer **3**) immunostimulatory 3pRNA containing a distal thiol modifier (i.e., on the opposite end of the 5′ triphosphate group, the 5′ position of the complementary strand) was next assessed. Briefly, electrostatic complexes or core conjugates with polymers **1** or **3**, respectively, were created by combining polymer and thiolated 3pRNA at pH 4 before raising to physiological pH, and corona conjugates were synthesized by addition of thiolated 3pRNA to preformed NPs of polymer **2**. These anticipated 3pRNA loading mechanisms for each polymer are illustrated in **Figure** **3**a. Upon addition of thiolated 3pRNA to polymers **2** and **3**, the mixture immediately turned yellow, which is indicative of the release of pyridine 2‐thione, the expected byproduct of thiol‐disulfide conjugation. Initially, polymer/3pRNA formulations were subjected to an electrophoretic mobility shift assay to assess the nature and degree of oligonucleotide loading for each polymer (Figure 3b; Figure S3, Supporting Information). Polymer and 3pRNA formulations containing 0.50 µg 3pRNA and prepared at a 10:1 polymer:RNA molar ratio were incubated with or without 12.5 USP units of heparin sulfate, a negatively charged biopolymer capable of competitively displacing 3pRNA from the cationic polymer, and/or 10 × 10^−3^ m tris(2‐carboxyethyl)phosphine (TCEP), a reducing agent that cleaves disulfide bonds between the 3pRNA and the polymer. For polymers **2** and **3**, which contain thiol‐reactive PDS groups, both heparin and TCEP were required to liberate 3pRNA from the polymer formulation, whereas polymer **1** released 3pRNA cargo while only in the presence of heparin. This result demonstrates that thiol‐modified 3pRNA can be covalently conjugated to both the corona‐forming (e.g., polymer **2**) or core‐forming (e.g., polymer **3**) blocks of the polymer carrier. Notably, despite conjugation to a preformed micelle, heparin is still required for full displacement of 3pRNA from polymer **2**, indicating that the cationic moieties of preformed micelles are sufficiently accessible to electrostatically interact with 3pRNA. Next, we sought to determine the minimum number of polymer chains per 3pRNA molecule required to achieve maximum loading (Figure 3c,d). To do this, we first quantified 3pRNA complexation by polymer **1** complexes using a RiboGreen exclusion assay and found that a minimum required polymer:3pRNA molar ratio (pol:3pRNA) of ≈5:1 is required to achieve maximum 3pRNA loading (Figure 3c). For 3pRNA‐polymer conjugates, we measured degree of conjugation using agarose gel electrophoresis and densitometric quantification of the free, unbound 3pRNA following incubation with heparin (but not TCEP) at varying pol:RNA ratios and found that a minimum pol:RNA ratio between ≈0.5 and 1 is required to achieve maximum conjugation (Figure 3d). Notably, maximum conjugation appears to be ≈80%, which is consistent with the measured number of thiols per oligo (Measure‐IT Thiol Assay kit, Invitrogen) and the predicted value based on oligo sequence length and the estimated coupling efficiency for the oligonucleotide synthesis process.^[^ ^52^ ^]^ Additionally, the size, polydispersity, and zeta potential of polymer/3pRNA formulations were tested at varying pol:3pRNA ratios and all formulations were found to have mean diameters ranging from 25 to 75 nm, with minimum dispersities (PDI < 0.4) observed in the pol:RNA window of 5–10, and a near‐neutral zeta potential (Figures 3e,f; Figure S4, Supporting Information). NP size decreased modestly with increasing pol:3pRNA ratio for all polymers, suggesting that the number of 3pRNA molecules per NP can influence how tightly polymer chains can pack together during the NP self‐assembly process, potentially by allowing more hydrophobic moieties in the core‐forming blocks to interact with each other (Figure 3e). Further, polymers **1** and **3** showed consistently low PDI (<0.3) at all tested pol:RNA ratios, unlike polymer **2**, which had a significantly higher PDI at most tested ratios (Figure 3f). This is likely due to minor degrees of crosslinking between polymer **2** NPs by spontaneous disulfide bonding, or perhaps by electrostatic attractions between corona conjugated 3pRNA and NP cores that draw particles together. The zeta potential for all three polymer/3pRNA formulations was near zero for all tested pol:3pRNA ratios (Figure S4, Supporting Information). Collectively, these data indicate that covalent conjugation of 3pRNA to the polymer backbone minimizes the required quantity of carrier to fully load 3pRNA while yielding nanoparticles with desirable physicochemical properties for in vivo administration. In particular, charge‐neutral particles below ≈100 nm in diameter are ideal candidates for increasing RNA half‐life, exploiting the enhanced permeation and retention effect for cancer therapy, and accessing draining lymph nodes following vaccine administration.^[^ ^42^, ^53^, ^54^, ^55^, ^56^, ^57^ ^]^

### 2.3 Covalent Conjugation Enhances 3pRNA Activity and Uptake in vitro

**Figure 4:**
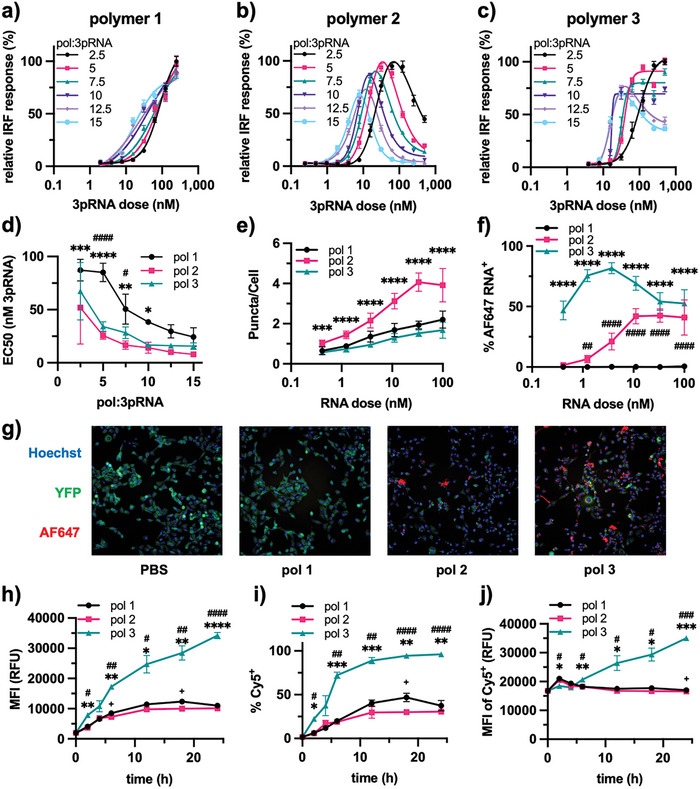
In vitro evaluation of RIG‐I activation, endosomal escape, and cellular RNA uptake by 3pRNA/polymer nanoparticles. a–c) Dose‐response curves for induction of IRF signaling in A549‐Dual reporter cells at indicated 3pRNA doses when a) complexed to polymer 1, b) conjugated to polymer 2, or c) conjugated to polymer 3 at indicated polymer to 3pRNA (pol:3pRNA) molar ratios and concentrations. *n* = 4 for each point. Data shown are representative of three independent experiments. IRF: Interferon Regulatory Factor. d) Calculated half‐maximal effective concentrations (EC_50_) of each formulation shown in a‐c) as a function of pol:3pRNA ratio. Comparisons made by 2‐way ANOVA with Dunnett's multiple comparisons test. Polymer **1** served as the control and each dose constitutes one family. *n* = 3 for all points. Asterisks (*) indicate comparisons between polymer **1** and polymer **2**. Hashes (#) indicate comparisons between polymer **1** and polymer **3**. *, ^#^ *P* < 0.05. ** *P* < 0.01. ****, ^####^ *P* < 0.0001. e) Enumerated fluorescent puncta per cell of Gal8‐YFP engineered MDA‐231 cells treated with indicated polymer/5′‐AlexaFluor‐647 (AF647) RNA formulations at pol:RNA = 10. *** *P* = 0.0008 and **** *P* < 0.0001. All comparisons shown are between polymers **2** and **3**. *n* = 12 for all points. f) Percentage of cells positive for AF647 in the experiment shown in e). The gating strategy to identify Cy5^+^ cells is shown in Figure S8 (Supporting Information). Asterisks (*) indicate comparisons between polymers **1** and **3**. Hashes (#) indicate comparisons between polymers **1** and **2**. ^##^ *P* = 0.008 and ****, ^####^ *P* < 0.0001. **g)** Representative merged images from the experiment shown in e,f) at an RNA dose of 3.70 nM (≈2 µg mL^−1^ polymer). Hoechst: nuclear stain. YFP: yellow fluorescent protein. AF647: AlexaFluor 647. e–g) Data shown are representative of two independent experiments. h) Flow cytometric analysis of median fluorescence intensity (MFI) of cells treated with 5′‐Cy5 RNA formulated with indicated polymer (pol:RNA = 10) after incubation for indicated duration. i) Percentage of cells positive for Cy5 (% Cy5^+^) in h). j) MFI of the Cy5^+^ subset of cells as shown in i). h,i,j) *n* = 3 independent formulations for each time point. Addition signs (+) indicate comparisons between polymer **1** and polymer **2**, asterisks (*) indicate comparisons between polymer **1** and polymer **3**, and hashes (#) indicate comparisons between polymer **2** and polymer **3**. ^+^, ^#^, * *P* < 0.05. **, ^##^ *P* < 0.01. ***, ^###^ *P* < 0.001. ****, ^####^ *P* < 0.0001. e,f,h,i,j) Comparisons shown are by 2‐way ANOVA with Tukey's multiple comparisons test. All data shown are mean +/−SD.

Next, we tested the immunostimulatory activity of polymer/3pRNA formulations at varying pol:3pRNA ratios in vitro in an A549‐Dual reporter cell line that expresses a secreted luciferase under control of a minimal ISG promoter (**Figure** **4**a–d). By establishing dose‐response curves and determining half‐maximal effective concentration (EC_50_) values, the relative potency of each formulation for inducing an IFN‐I response can be determined. As expected, all three polymers were effective at stimulating an IFN‐I response via delivery of 3pRNA, with increased activity observed at higher pol:3pRNA ratios (Figures 4a–c) which is consistent with previous reports using other cationic materials and nucleic acid cargo.^[^ ^58^, ^59^, ^60^, ^61^, ^62^, ^63^ ^]^ Moreover, higher pol:3pRNA ratios increase the dose of polymer relative to the dose of 3pRNA, which serves to further increase the dose‐dependent endosomal escape efficiency observed with these polymers (Figure 2i). As cytosolic delivery of 3pRNA cargo is a direct consequence of endosomal escape, it follows that higher pol:3pRNA ratios are more effective at stimulating IFN‐I response. In contrast to 3pRNA NPs formulated with polymers **1** and **3**, the cells treated with 3pRNA‐polymer **2** conjugates show appreciable dose‐ and pol:3pRNA ratio‐dependent cytotoxicity, which is consistent with the cytotoxicity data shown in Figure 2g and the decrease in measured IFN‐I response at higher doses as shown in Figure 4b (Figure S5, Supporting Information). This dose‐ and pol:3pRNA ratio‐dependent cytotoxicity effect was also observed in cells treated with 3pRNA‐polymer **3** conjugates, although to a much lesser extent (Figure S5c, Supporting Information). Collectively, these data support the possibility that residual PDS groups on the surface of polymer **2** NPs may facilitate cytotoxic levels of polymer uptake into cells up by binding to cell surface thiols. The EC_50_ values for IFN‐I activation of all polymer/3pRNA formulations are plotted in Figure 4d, where it is evident that formulations prepared using polymers **2** and **3**, in which 3pRNA is covalently conjugated to polymer, are more potent than those assembled via only electrostatic interactions with polymer **1** at pol:RNA ratios of 10 and below. While the maximum measured IFN‐I response of cells treated with 3pRNA conjugates of polymers **2** and **3** show a significant decrease with increasing pol:3pRNA ratio when compared to the IFN‐I response of those treated with 3pRNA formulated with polymer **1**, the magnitude of this effect is modest (Figure S6, Supporting Information). Furthermore, the increased cytotoxicity of 3pRNA formulated with polymers **2** and **3** may explain the decrease in the maximum IFN‐I response and IFN‐I activity at higher doses, as nonviable cells are unable to contribute to an IFN‐I response. These data indicate that covalent conjugation achieves higher or equivalent 3pRNA‐mediated RIG‐I activation potency using lower quantities of carrier material with no or minimal sacrifice in maximum efficacy. For polymers **2** and **3**, increasing the pol:RNA ratio beyond ≈10:1 resulted in minimal gains in potency, motivating the selection of a 10:1 pol:3pRNA ratio for all subsequent investigations.

After selecting a common pol:RNA ratio at which to compare all three polymers, we next sought out to further characterize the differences in activity by examining the intracellular delivery of RNA cargo. To investigate this, polymers were loaded with 5′‐AlexaFluor647 (AF647) RNA, in which the 5′ppp moiety was replaced with AF647, to simultaneously evaluate intracellular RNA uptake in concert with the Gal8 recruitment assay used to assess endosomal escape (Figures 4e–g). As anticipated, endosomolytic events (i.e., Gal8 puncta per cell) scaled with polymer/RNA dose for all polymers, with polymer **2** causing the most endosomolysis and polymers **1** and **3** displaying similar levels of activity (Figure 4e). The increased endosomolysis of cells treated with conjugates formulated with polymer **2** is consistent with its elevated cytotoxicity (Figure S5b, Supporting Information), as endosomal disruption has been found to promote cell death.^[^ ^64^, ^65^, ^66^ ^]^ Surprisingly, the fraction of AF647^+^ cells following incubation with the polymer **1** formulation was nearly zero at all tested doses, whereas those of the polymer **2** and **3** formulations were increased relative to polymer **1** at nearly all doses (Figure 4f). To investigate this further, we examined the mean cell‐average integrated intensity of AF647 in each replicate and detected a small subset of cells treated with formulations of AF647‐RNA and polymer **1** with an MFI comparable to cells treated with polymer **2** and **3** formulations, but the majority of cells still showed no uptake (Figure S7, Supporting Information). Representative images of merged signals from this experiment at an RNA concentration of 3.70 × 10^−9^ m (≈2 µg mL^−1^ polymer) are displayed in Figure 4g. The low uptake of AF647‐labeled RNA measured in the MDA‐MB‐231‐derived cell line was in direct contrast with the dose‐dependent immunostimulatory activity observed with 3pRNA/polymer **1** NPs in the A549‐Dual reporter cells (Figure 4a), potentially suggesting that the extent to which covalent conjugation of RNA increases cellular uptake is cell‐type dependent.

To further interrogate the influence of covalent conjugation on RNA uptake, polymers were loaded with 5′Cy5‐labeled RNA and incubated at 20 × 10^−9^ m RNA with A549 lung carcinoma cells for different durations and the relative degree of uptake was analyzed by flow cytometry (Figure 4h–j; Figure S8, Supporting Information). A time‐dependent increase in the measured median fluorescence intensity (MFI, Figure 4h) and percentage of Cy5^+^ cells (Figure 4i) was observed for all three polymers, with polymer **3** NPs promoting significantly more RNA uptake than NPs of polymer **1** or polymer **2**, which had very similar uptake profiles. Interestingly, only polymer **3** resulted in a continuous increase in the MFI of Cy5^+^ cells over the course of 24 h (Figure 4j), suggesting that conjugation to the core‐forming block promotes continued delivery of RNA payload to cells that have already endocytosed NPs (i.e., are already Cy5^+^). Collectively, these data demonstrate an interplay between endosomal escape efficiency and the magnitude and/or kinetics of cellular uptake, as well as suggest that covalent conjugation to the core‐forming block may have benefits over conjugation to the corona‐forming block.

### 2.4 Covalent Conjugation of 3pRNA to Polymer Nanocarrier Confers Nuclease Resistance and Serum Stability

**Figure 5:**
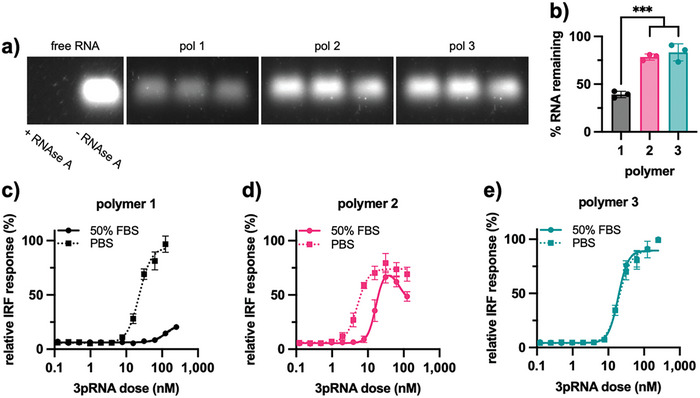
Evaluation of nuclease and serum stability of polymer/3pRNA formulations. a) Electrophoretic mobility shift assay of indicated 3pRNA formulations following RNase A challenge. b) Densitometric quantification of remaining RNA bands in a) as shown for indicated polymer/RNA formulations with polymer:3pRNA ratio of 10. *n* = 3 independent formulations each. Comparisons by ordinary one‐way ANOVA with Tukey's multiple comparisons test. c–e) Dose–response curves for induction of IRF signaling in A549‐Dual reporter cells at indicated 3pRNA doses when c) complexed to polymer **1**, d) conjugated to polymer **2**, or e) conjugated to polymer **3** at indicated polymer:3pRNA molar ratios following serum challenge. *n* = 4 for each point. Data shown are representative of two independent experiments. IRF: Interferon Regulatory Factor. All data shown are mean +/−SD.

We hypothesized that the discrepancies between the endosomolytic activities and delivery efficiencies of the polymer/3pRNA formulations could be at least partially explained by differences in the nuclease or serum resistances of the resultant NPs. To investigate this, polymer/3pRNA formulations were incubated with an excess of RNase A at physiological pH and temperature before being subjected to the electrophoretic mobility shift assay employed previously (**Figure** **5**a; Figure S9, Supporting Information). Interestingly, formulations with polymers **2** and **3** show undegraded 3pRNA fractions comparable to the fraction of 3pRNA conjugated to the polymer (Figures 5b and 3d, respectively). This contrasts with electrostatic complexes prepared with polymer **1**, where over half of RNA cargo is degraded despite a comparable degree of loading (Figure 3c). These data indicate that covalent conjugation of 3pRNA cargo to the polymer nanocarrier is important for improving nuclease resistance. Surprisingly, the choice of conjugation to the corona of a micelle or the core of an electrostatic complex did not have a significant effect on nuclease resistance.

To interrogate further, we employed a serum stability assay, in which polymer/3pRNA NP formulations were incubated 50% fetal bovine serum (FBS) or PBS and incubated at 37 °C for 4 h before testing immunostimulatory activity in vitro (Figures 5c–e). The formulation made with polymer **1** (electrostatic only) lost nearly all activity (Figure 5c), while the EC_50_ of the formulation made with polymer **2** (corona conjugate) increased by a factor of about 3 after serum exposure relative to incubation in PBS (EC_50_’s = 15.48 × 10^−9^ and 5.438 × 10^−9^ m, respectively, Figure 5d). Strikingly, and in contrast to polymers **1** and **2**, the activity of 3pRNA formulated with polymer **3** was not affected by incubation in 50% serum (Figure 5e). This demonstrates that despite being not particularly important for imparting nuclease resistance, the chosen site of conjugation on the nanocarrier is important for maintaining serum stability. We postulate that 3pRNA conjugated to the core of a nanocarrier may be less accessible to serum components than 3pRNA conjugated to the corona, and/or that core conjugates harness both electrostatic and covalent interactions to increase particle stability in serum. Additionally, it cannot be discounted that residual PDS groups on the surface of NPs prepared with polymer **2** could react with free thiols present on serum proteins, possibly resulting in reduced stability.^[^ ^50^ ^]^ Taken together, these data indicate that covalent loading of 3pRNA into polymer nanocarrier confers additional durability to the resulting formulation in situ, with conjugation to the core‐forming block being particularly advantageous. Furthermore, the reduced RNase resistance of 3pRNA loaded into polymer **1** and reduced serum stability of RNA loaded into polymers **1** and **2** could potentially explain the poor cellular uptake of RNA observed when formulated with these polymers and the continuously increasing uptake of RNA formulated to polymer **3** (Figures 4f,h–j).

### 2.5 Immunostimulatory Activity of 3pRNA is Site‐specific and Linker Chemistry‐Dependent with Respect to Thiol Attachment Chemistry

**Figure 6:**
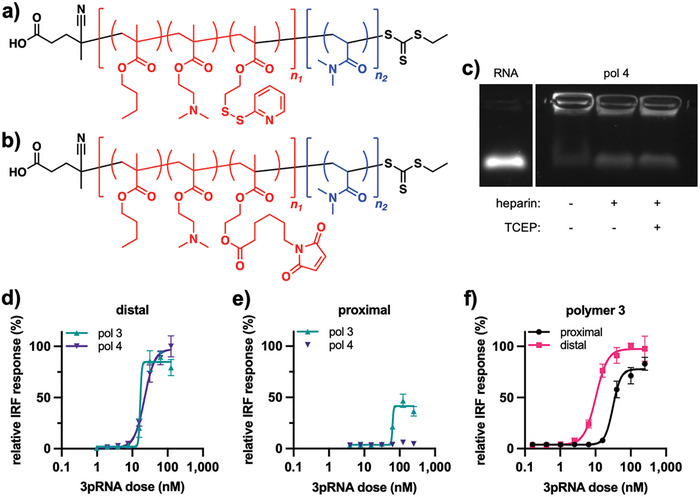
Analysis of thiol site‐specificity and linker chemistry dependencies on the immunostimulatory activity of polymer/3pRNA conjugates. a,b) Structures of polymers 3 and 4. a) Polymer 3 contains PDS groups that undergo thiol exchange reactions to form a cleavable disulfide bond with thiol‐modified RNA while, b) Polymer 4 contains maleimide groups that react with thiol‐modified RNA to form a stable thioether bond. c) Electrophoretic mobility shift assay demonstrating conjugation of 3pRNA to polymer 4. d,e) Dose–response curves for induction of IRF response following treatment of A549‐Dual reporter cells at indicated 3pRNA doses with indicated polymers of d) distal and e) proximal conjugates. *n* = 4 for each point. Data are representative of three independent experiments. f) Dose–response curves following treatment of A549‐Dual reporter cells at indicated 3pRNA doses loaded into polymer 3 with indicated oligonucleotide orientation. *n* = 4 for each data point. All data shown are mean +/−SD.

Based on its potent immunostimulatory activity and resistance to nuclease and serum degradation, we selected polymer **3** as the best candidate to further probe the dependence on thiol placement and linker chemistry, which we identified as potentially important variables in our previous report.^[^ ^43^ ^]^ Here, we refer to thiol placement at the 5′ end of the strand complement to that with the 5′ppp moiety as “distal,” which is the site of conjugation used for all studies described above. Thiol placement instead at the 3′ end of the complement strand is referred to herein as “proximal.” These contrasting orientations of attachment chemistry are illustrated in Figure 1f. Additionally, to evaluate the importance of the disulfide bond cleavability, polymer **4** was synthesized as a cleavage‐resistant analog of polymer **3**, substituting pendant PDS groups for pendant thiol‐reactive maleimide groups such that the bioreducible disulfide bond was replaced with a stable thioether bond (**Figures** **6**a,b; Table S1, Supporting Information). As anticipated, when subjected to the denaturing electrophoretic mobility shift assay described above, conjugates of polymer **4** were resistant to TCEP‐mediated 3pRNA liberation (Figure 6c; Figure S10, Supporting Information), which is in direct contrast to the reductive cleavability of 3pRNA conjugates of polymers **2** and **3** (Figure 3b).

We then compared the immunostimulatory activity of proximal and distal 3pRNA conjugates prepared using polymers **3** and **4** in A549‐Dual reporter cells at a pol:3pRNA ratio of 10 (Figure 6d,e). Surprisingly, distal conjugates of polymer **4** were nearly identical in activity and uptake to polymer **3** (Figure 6d; Figure S11, Supporting Information), suggesting that 3pRNA may not have to be released from the polymer if a blunt ended dsRNA with a 5′ triphosphate moiety is accessible to bind RIG‐I. This behavior is consistent with our previous work, in which conjugation of a 5 kDa mPEG to the distal site via thioether linkage did not interfere with immunostimulatory activity, though this finding is still somewhat unexpected given the approximately ten‐times higher MW of the polymers used here (Table S1, Supporting Information).^[^ ^43^ ^]^ Based on this finding, it is possible that other covalent attachment chemistries can be leveraged for distal ligation, such as sulfonyl fluoride exchange, azide‐alkyne cycloaddition, or *trans*‐cyclooctene tetrazine inverse‐electron‐demand Diels‐Alder ligation.^[^ ^67^, ^68^, ^69^ ^]^ As expected, proximal conjugates of polymer **3**, but not polymer **4**, were capable of stimulating RIG‐I, which is also consistent with our previous findings demonstrating that stable conjugation of mPEG to a proximal thiol blocks RIG‐I binding (Figure 6e).^[^ ^43^ ^]^ In another independent experiment, we compared the activity of proximal and distal disulfide conjugates of polymer **3**, we found that the proximal conjugates had reduced activity despite an equivalent activity between unthiolated 3pRNA and proximally thiolated 3pRNA (Figure 6f, ref.[43]). We suspect that this might be related to an incomplete release of proximally ligated 3pRNA, which would be required for RIG‐I activation. While further investigation of this behavior is necessary, these data support the possibility that the magnitude and kinetics of RIG‐I activation may be modulated via control of proximal site linker chemistry and cleavage rate.

### 2.6 Distal Conjugation of 3pRNA to Polymer Nanocarrier Improves RIG‐I Activation In Vivo

**Figure 7:**
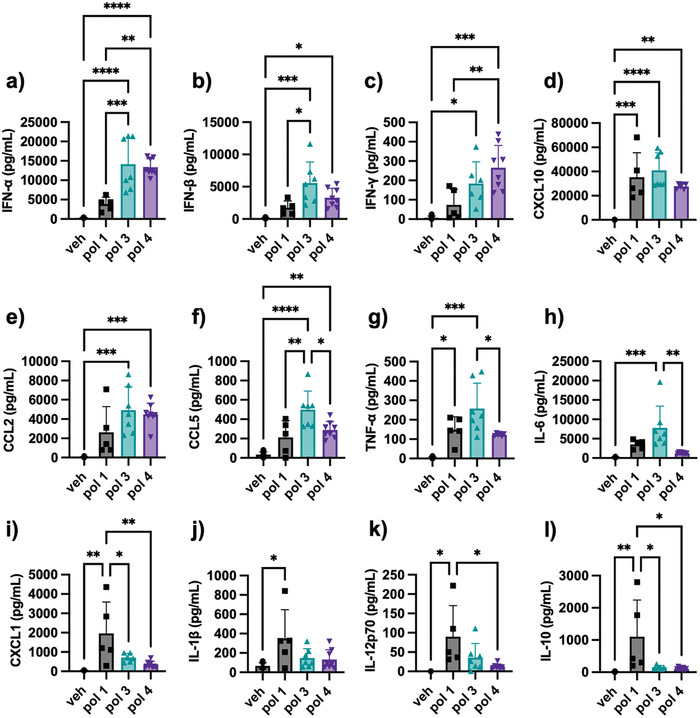
In vivo immunostimulatory activity of polymer/3pRNA formulations. Plasma concentrations of indicated cytokines 4 h after intravenous administration of polymer/3pRNA nanoparticles at 1.2 mg 3pRNA kg^−1^ body mass formulated with indicated polymer. Comparisons made by one‐way ANOVA with Tukey's multiple comparisons test. *n* = 6 (vehicle), *n* = 5 (polymer **1**), *n* = 7 (polymer **3**), *n* = 8 (polymer **4**). * *P* < 0.05. ** *P* < 0.01. *** *P* < 0.001. **** *P* < 0.0001. All data shown are mean +/‐ SD.

We next evaluated the ability of 3pRNA complexed to polymer **1** or distally conjugated to polymers **3** or **4** to activate RIG‐I in vivo. We elected to exclude polymer **2** due to the reduced benefits of corona conjugation (Figures 5d,e) and the increased cytotoxicity associated with this carrier (Figure 2g; Figure S5b, Supporting Information). First, while it has been previously demonstrated that replacing the 3p moiety with an OH group eliminates RIG‐I binding,^[^ ^2^ ^]^ we nonetheless first compared the in vivo activity of polymer **3** conjugates prepared using 3pRNA or negative control OH‐RNA, demonstrating and confirming that the 3p group was essential for activation of an IFN‐I response in mice (Figure S12, Supporting Information). Next, 3pRNA was formulated with polymers **1**, **3**, and **4** at a pol:3pRNA ratio of 10:1 as described above using distally thiolated 3pRNA. Polymer/3pRNA formulations were buffer exchanged into sterile 5% D‐glucose and injected into healthy, 8‐week‐old female mice by retro‐orbital injection at a dose corresponding to 1.2 mg 3pRNA kg^−1^ body mass. 4 h later, the mice were euthanized, and their blood was collected for quantification of plasma IFN‐Is and other antiviral cytokines and chemokines. We found that all three polymer/3pRNA treatments exhibited in vivo activity as indicated by elevation of one or more of the antiviral cytokines/chemokines assayed (**Figure** **7**). Importantly, polymer/3pRNA core conjugates formulated using polymers **3** and **4** resulted in significantly increased plasma concentrations of IFN‐α and IFN‐β – the “signature” cytokines associated with RIG‐I activation with important antiviral and antitumor functions – relative to vehicle control and 3pRNA electrostatically complexed with polymer **1** (Figure 7a,b)**.^[^** ^3^ ^]^ Additionally, polymers **3** and **4** elevated the plasma concentrations of IFN‐γ, C‐X‐C motif chemokine ligand 10 (CXCL10), C‐C motif chemokine ligand 2 (CCL2), and CCL5 (Figure 7c–f). While the response to 3pRNA conjugates prepared with polymers **3** and **4** was generally equivalent for measured cytokines, polymer **3** resulted in significantly elevated levels of tumor necrosis factor α (TNF‐α) and interleukin 6 (IL‐6, Figure 7g,h) relative to polymer **4**, suggesting that the linker chemistry used to covalently load 3pRNA can potentially affect the nature or kinetics of the downstream immune response following activation of RIG‐I. Interestingly, while administration of 3pRNA loaded into polymer **1** by electrostatic complexation increased the plasma concentration of CXCL10 and TNF‐α to a level comparable to conjugates made with polymer **3** and **4** (Figure 7d,g), this was also associated with a significant increase in CXCL1, IL‐1β, IL‐12p70, and IL‐10 (Figure 7i–l), which were not significantly elevated by 3pRNA covalently loaded into polymers **3** or **4**. Overall, 3pRNA loaded covalently into polymers **3** and **4** performed fairly consistently with each other despite differing attachment chemistry, and, importantly, both conjugates drastically outperformed 3pRNA loaded electrostatically into polymer **1** in terms of the magnitude and specificity of the antiviral immune response. Given the differential expression kinetics and serum half‐lives of these cytokines, and considering the complex network of signaling events that contribute to the concentration of any chosen cytokine at a given time point, considerably more investigation is necessary to further interrogate the innate immune response elicited by 3pRNA formulations prepared with each polymer and to determine if the response can be tuned via control of carrier properties and 3pRNA loading chemistry.

## 3 Conclusion

Engineering of polymeric nanocarriers holds potential for overcoming critical delivery barriers limiting the development of 3pRNA therapeutics and their translation into the clinic. However, there has been relatively little investigation into the development of polymeric delivery systems for 3pRNA, an oligonucleotide therapeutic whose distinctive structure and properties impose unique carrier design requirements. Using conjugatable and nonconjugatable analogs of a polymeric nanocarrier that we had previously designed for electrostatic complexation of 3pRNA (4‐50), we have demonstrated that the loading efficiency and immunostimulatory activity of 3pRNA can be substantially enhanced if covalently conjugated to the polymer backbone. Importantly, we also found that covalent conjugation of 3pRNA protected against nuclease degradation and serum destabilization, particularly when the 3pRNA was ligated to the core‐forming, endosomolytic block. Consequently, systemic administration of nanoparticles formed with core conjugated 3pRNA enhanced type‐I IFN production to a greater extent than those assembled using only electrostatics. Notably, core conjugation still enhanced activity despite employing a polymer with reduced endosomolytic activity, reflecting a balance of nucleic acid stability, cellular uptake, and endosomal escape conferred by core conjugation that may also benefit other oligonucleotide therapeutics (e.g., siRNA, miRNA). Further, we observed an interplay between the cleavability of the 3pRNA‐polymer bond and the site‐specificity of the thiol modifier, as conjugation to the distal end of 3pRNA is permissive to stable ligation via a thioether bond, whereas proximal ligation requires a cleavable linkage that allows liberation of 3pRNA from the carrier, which is consistent with our previous report.^[^ ^43^ ^]^ These findings have potential implications for the engineering of carrier platforms that enable spatiotemporal control of RIG‐I activation through design of environmentally responsive ligation chemistries and/or bond cleavage kinetics. Overall, these studies constitute the first description of covalent 3pRNA‐nanocarrier conjugates and highlight how rational integration of covalent 3pRNA conjugation to polymer backbones is a promising strategy for the optimization of future nanocarrier formulations of 3pRNA with potential clinical applications.

## 4 Experimental Section

### Materials

Oligonucleotide synthesis reagents were purchased from BioAutomation (Irving, TX) and Glen Research (Sterling, VA). 2′‐pivaloyloxymethyl amidites were purchased from ChemGenes (Washington, MA). Ethanethiol, sodium hydride (60% in oil), carbon disulfide, molecular iodine, 4,4′‐azobis(4‐cyanovaleric acid), butyl methacrylate (BMA), 2‐(dimethylamino)ethyl methacrylate (DMAEMA), *N,N*‐dimethylacrylamide (DMA), cysteamine hydrochloride, (meth)acryloyl chloride, azobisisobutyronitrile (AIBN), organic solvents, D‐glucose (BioXtra), tris(2‐carboxyethyl)phosphine (TCEP), heparin sodium salt (from porcine intestinal mucosa, ≥180 USP units mg^−1^), Triton‐X 100, and oligonucleotide triphosphorylation and deprotection reagents were obtained from Sigma‐Aldrich (St. Louis, MO). 2,2‐Dithiodipyridine and β‐mercaptoethanol were purchased from Oakwood Chemical (Estill, SC). V‐70 azo initiator was procured from FUJIFILM Wako Chemicals (Richmond, VA). Fetal bovine serum (FBS), heat‐inactivated fetal bovine serum (HI‐FBS), phosphate‐buffered saline (PBS, 1×), penicillin/streptomycin solution, and Dulbecco's Modified Eagle Medium (DMEM) and opti‐MEM reduced serum media were procured from Gibco (Grand Island, NY). 0.5 m disodium *N*,*N*,*N* *’*,*N* *’*‐ethylenediamine tetraacetic acid (EDTA) solution, 1.0 m (4‐(2‐hydroxyethyl)−1‐piperazineethanesulfonic acid) (HEPES) solution, and molecular biology‐grade water were purchased from Corning (Corning, NY). Lipofectamine 2000 was acquired from Invitrogen (Carlsbad, CA). Chromatography‐grade glacial acetic acid was obtained from Fisher Scientific (Hampton, NH). 3′‐and 5′‐disulfide‐modified antisense strand RNA (sequence: 5′‐AUA GGC GUA UUA UAC GCG AUU AAC G‐3′), as well as 5′‐nonphosphorylated sense strand, 5′‐AlexaFluor 647‐tagged sense strand (sequence: 5′‐UAU CCG CAU AAU AUG CGC UAA UUG C‐3′), and unmodified antisense strand RNAs were purchased from Integrated DNA Technologies (Coralville, IA). Monomers 2‐(pyridin‐2‐yldisulfaneyl)ethyl methacrylate (PDSEMA) and *N*‐(2‐(pyridin‐2‐yldisulfaneyl)ethyl)acrylamide (PDSEAm) were synthesized as described elsewhere.^[^ ^70^, ^71^ ^]^ All other reagents were of analytical grade from Sigma‐Aldrich.

### Synthesis and Purification of 5′ Triphosphate RNA (3pRNA)

3pRNA (seq: 5′‐ppp‐CGU UAA UCG CGU AUA AUA CGC CUA U‐3′) was synthesized on a MerMade 12 DNA‐RNA synthesizer (BioAutomation) as described previously.^[^ ^72^, ^73^, ^74^ ^]^ The 3pRNA was deprotected using ammonium and purified by polyacrylamide gel electrophoresis before further purity analysis by mass spectrometry (Novatia, LLC).

### Synthesis of 5′Cy5 RNA

RNA (seq: Cy5*C*G*U UAA UCG CGU AUA AUA CGC CU*A*U, asterisks indicating phosphorothioate bonds) was synthesized at 1 µmole scale on a MerMade 12 DNA synthesizer. 5‐Ethylthio‐1H‐Tetrazole (0.25 m in acetonitrile) was used as activator, 0.02 m Iodine in Tetrahydrofuran/Pyridine/Water (70:20:10) was used as the oxidizer, 0.05 m Sulfurizing Reagent II in Pyridine/Acetonitrile was used as the sulfurizing agent, and a solution of 3% α‐dichloroacetic acid in methylene chloride (DCM) was used as the deblock solution. Oligonucleotides were grown on 1000 Å CPG functionalized with Unylinker (≈42 µmol g^−1^). 2′‐TBDMS and 5′ Cy5 phosphoramidite bases were dissolved in acetonitrile at a concentration of 0.15 m with a coupling time of 11 min for each base. The nucleobase protecting groups were removed with concentrated aqueous ammonium hydroxide for 48 h at room temperature. Deprotection of the TBDMS group was achieved with DMSO:NEt_3_•3 HF (4:1) solution (200 µL) at 65 °C for 3 h. The RNA oligonucleotide was then immediately purified on a Glen‐Pak RNA cartridge (Glen Research) according to the manufacturer's instructions. The purified oligo was lyophilized and resuspended in RNase‐free water containing 1 × 10^−3^ m EDTA. Molecular weight and purity were confirmed using Liquid Chromatography‐Mass Spectrometry (LC‐MS) analysis on a ThermoFisher LTQ Orbitrap XL Linear Ion Trap Mass Spectrometer. Chromatography was performed using a Waters XBridge Oligonucleotide BEH C18 Column using a linear gradient from 85% A (16.3  × 10^−3^ m triethylamine + 400  × 10^−3^ m hexafluoroisopropanol) to 100% B (methanol) at 45 °C.

### Synthesis of Chain Transfer Agent (CTA) 4‐cyano‐4‐(((ethylthio) Carbonothioyl)thio) Pentanoic Acid (ECT)

ECT was synthesized as previously described with minor modifications.^[^ ^75^ ^]^ Initially, ethanethiol (6.3 g, 100 mmol) was added dropwise to a suspension of sodium hydride (60% in oil, 4.2 g, 105 mmol) in 150 mL diethyl ether with vigorous stirring at room temperature, which was continued until the evolution of gas was no longer observed. After the evolution of gas bubbles ceased, carbon disulfide (8.0 g, 105 mmol) was added to the reaction and allowed to stir for 30 minutes to yield sodium *S*‐ethyl trithiocarbonate as a yellow precipitate, which was collected by filtration, washed with 50 mL diethyl ether, and resuspended in 100 mL of fresh diethyl ether. Then, molecular iodine (13.2 g, 52 mmol) was added portion‐wise to this resuspension, which was then stirred for 1 hour at room temperature. Sodium iodide byproduct was removed by filtration and the remaining solution was washed three times with equal volumes of saturated aqueous sodium thiosulfate, once with water, and once with brine. This washed solution was then dried over anhydrous sodium sulfate. Solvent was then removed by rotary evaporation to yield bis‐(ethylsulfanylthiocarbonyl) disulfide as a dark yellow‐orange oil (13.7 g, 50 mmol, quantitative).

Bis‐(ethylsulfanylthiocarbonyl) disulfide (5.3 g, 19.3 mmol) and 4,4′‐azobis(4‐cyanovaleric acid) (8.1 g, 28.9 mmol, 1.5 eq.) were dissolved in 200 mL of ethyl acetate and were refluxed vigorously for 18 h under a nitrogen atmosphere. The solvent was then removed by rotary evaporation and the resulting crude oil was purified by silica gel chromatography (1:1 hexane:ethyl acetate (v:v) supplemented with + 0.1% glacial acetic acid (v:v), ECT *R*_f_ = 0.2, *R*_f_ = 0.9 is bis‐(ethylsulfanylthiocarbonyl) disulfide). The fractions containing ECT were pooled, and the elution solvent was removed by rotary evaporation, using a few additions of 10 mL of toluene near the end of the evaporation process to coevaporate residual acetic acid. The remaining material was dried in vacuo for 1 week and ground to afford the title product as a bright yellow powder (3.9 g, 77%).

### Synthesis and Characterization of Polymers 1–3

The general procedure for polymer synthesis by reversible addition‐fragmentation chain‐transfer (RAFT) polymerization of the polymers in this study is described as follows. Polymerization inhibitors were removed from commercially obtained monomers by simple gravity chromatography through a column of basic activated alumina (Brockmann type I) immediately before use. Monomer(s), CTA, and radical initiator were dissolved in toluene, an aliquot was taken as a reference for ^1^H NMR analysis (Bruker AV 400), and the polymerization mixture was purged with nitrogen gas for 30 min before placing the reaction mixture in an oil bath at indicated temperature for 18 h. The initial CTA:initiator ratio was 20:1 for all reactions. M_0_:(macro‐)CTA ratios, initial monomer compositions, choice of (macro‐)CTA, choice of initiator, mass fraction of toluene, and reaction temperature for all polymerizations are summarized in Table S2 (Supporting Information).

After polymerization, the reactions were terminated by briefly purging the crude mixtures with air, and monomer conversion was determined by comparing the proportional peak intensities of vinyl peaks and ester peaks from ^1^H‐NMR before and after polymerization. Polymers were isolated by precipitation into pentane and subsequent redissolution into acetone and reprecipitation into fresh pentane for a total of five precipitations. Polymers were then dried in vacuo for 3 d. Polymer compositions were also determined by ^1^H NMR spectroscopy of the pure products. Gel permeation chromatography was performed using 10 × 10^−3^ m LiBr in dimethylformamide as a mobile phase to elute through two TSKGel Alpha columns in series (2500 and 3000, Tosoh Biosciences) and comparing to pMMA standards. GPC traces can be found in Figure S1 (Supporting Information) and example ^1^H‐NMR spectra of polymers **1**, **2**, and **3** can be found in Figures S13–S15 (Supporting Information), respectively.

An example synthesis using polymer **2** as reference is as follows. After filtering monomers through simple alumina columns, ECT (26.6 mg, 101 µmol), DMAEMA (1.99 g, 12.6 mmol), and BMA (1.80 g, 12.6 mmol) were combined in a 10 mL flask with 3.66 g toluene, 1.56 mg AIBN (as 156 mg of 1.00 wt% solution in toluene, 5.05 µmol), and a magnetic stirrer. The mixture was stirred until ECT was fully dissolved, and then the flask was sealed, gently purged with nitrogen gas for 30 min, and held at 75 °C for 18 h. The resulting mixture was precipitated five times into hexane and dried to yield polymer mCTA **2**. In a new flask, 716 mg dry mCTA **2** (20.3 µmol) was combined with 439 mg filtered DMA, 65.6 mg PDSEAm, 1.77 g toluene, and a magnetic stirrer. The flask was sealed and briefly placed in an unheated ultrasonic bath with periodic mixing to assist dissolution of mCTA **2** in the toluenic monomer mixture. When everything was completely in solution, 310 µg V‐70 (as 62 mg of 0.5 wt% solution in toluene, 1.0 µmol) was added and the mixture was thoroughly mixed before resealing and again gently purging with nitrogen gas for 30 min before holding at 40 °C for 18 h. Chain extension of DMAEMA‐containing mCTAs with PDS‐containing monomers (either already copolymerized into the mCTA or included as a chain extension monomer) tend to induce PDS crosslinking during the polymerization process at higher temperatures, necessitating an initiator with a lower temperature elevation requirement for chain extension (unpublished observations). The resulting mixture was purified by the same method as mCTA **2** to yield polymer **2** as a yellow solid.

### Synthesis of Maleimide‐modified Polymer 2

Polymer 2 was suspended in ethanol at 1  × 10^−3^ m and mixed at a 50:50 ratio with 5 × 10^−3^ m TCEP for one hour at room temperature. Samples were run through a 7k Zeba column to remove excess TCEP, then reacted with 5 × 10^−3^ m *N*‐ethylmaleimide overnight. The following day capped polymer was precipitated in ice cold pentane for five cycles spinning at 200 × *g* for 5 min each. The polymer was then dried for 48 h prior to use in vitro. ^1^H‐NMR of maleimide‐modified polymer **2** is available in Figure S16 (Supporting Information).

### Synthesis of Polymer 4

To synthesize maleimide‐functionalized polymer **4**, precursor polymer **4‐OH** was synthesized as a version of polymer **3** with 2‐hydroxyethyl methacrylate (HEMA) in place of PDSEMA using otherwise identical polymerization and purification conditions. A flask that had been freshly baked overnight at 120 °C was charged with a magnetic stir bar, polymer **4‐OH** (990 mg, 20 µmol, 244 µmol HEMA), 6‐maleimidohexanoic acid (771 mg, 3.65 mmol, 15 eq with respect to HEMA), dicyclohexyl carbodiimide (902 mg, 4.37 mmol, 18 eq), and a catalytic amount of *N,N*‐dimethylaminopyridine (1.5 mg, 0.05 eq). The solids were dissolved in 50 mL anhydrous DCM by gentle mixing and unheated sonication, and then the flask was sealed, the solution was gently purged with nitrogen gas for 30 min, and the mixture was stirred overnight at room temperature. The following day, insoluble dicyclohexylurea byproduct was removed by filtration of the reaction mixture through celite before removal of the solvent by rotary evaporation. The remaining mixture was redissolved in DCM (≈30 wt% solids), and the polymer was selectively precipitated by first diluting the polymer solution with diethyl ether (12.5 mL per 2 mL polymer + DCM solution) then the subsequent addition of hexane (3 mL per 5 mL ether) precipitated the desired product. The precipitation mixture was vortexed, the polymer was pelleted by centrifugation, and the supernatant was removed by decantation. The polymer was redissolved with minimal DCM and the precipitation process was repeated for 4 additional precipitations. The polymer pellet was then dried in vacuo for 3 days to yield polymer **4** as a light brown, crumbly solid. ^1^H‐NMR of polymer **4** is available in Figure S17 (Supporting Information).

### Formulation of and Characterization of Polymer Micelles

Aqueous solutions of self‐assembled diblock copolymer micelles were prepared by initially dissolving polymers in ethanol at 100 mg mL^−1^ and diluting by portionwise addition of a sterile‐filtered HEPES‐buffered glucose solution (HBG, 50 × 10^−3^ m HEPES, 5 × 10^−3^ m EDTA, 5% D‐glucose (w/v), pH 7.4) with gentle mixing until concentration of 10 mg mL^−1^ was reached. These 10 mg mL^−1^ aqueous solutions were diluted to 50 µg mL^−1^ in 1x PBS or 10 × 10^−3^ m citrate buffer (pH 4.2) for sizing measurements by dynamic light scattering (DLS) using a Malvern Zetasizer (Malvern, USA).

### Preparation of Thiolated RNAs

Before formulation of polymer/RNA complexes or polymer‐RNA conjugates, 5′‐triphosphate, 5′‐hydroxyl, or 5′‐fluorophore sense strand RNA and 5′‐ (distal) or 3′‐thiolated (proximal) antisense RNA (as shipped in protected disulfide form) were mixed in equimolar amounts and supplemented with DTT (100 × 10^−3^ m). This mixture was allowed to anneal by incubating at 95 °C for five minutes and slowly cooling to 4 °C over the course of 45 min. After completion, the annealed and deprotected thiol‐RNA was passed through two Zeba Spin Desalting Columns (7 kDa MWCO, Thermo Scientific, Waltham, MA) in series that were pre‐equilibrated with 1 × 10^−3^ m EDTA. Any remaining DTT was then removed by five consecutive rounds of centrifugal diafiltration (Amicon Ultra centrifugal filters, 10 kDa MWCO, MilliporeSigma, MA) with. The concentration of the resulting RNA was determined by absorbance at 260 nm using a Synergy H1 microplate reader (BioTek, Winooski, VT). Each batch of 5′ thiol RNA was tested using the fluorometric Measure‐IT Thiol Assay kit (Invitrogen) and they were consistently found to be ≈80% thiolated. The 3′ thiol RNA was tested using the same kit and was found to be > 95% thiolated.

### Preparation of Polymer 1/RNA Complexes, Polymer 3‐RNA Conjugates, and Polymer 4‐RNA Conjugates

Thiolated oligonucleotide was diluted to 1 mg mL^−1^ in a sterile‐filtered 10  × 10^−3^ m citrate + 1 × 10^−3^ m EDTA buffer (CE, pH 4.2) and polymers were diluted from their 100 mg mL^−1^ ethanolic stock solution directly to 25 mg mL^−1^ with the same CE buffer. These oligonucleotide and polymer solutions were mixed at ratios appropriate to achieve the indicated polymer:RNA molar ratios and incubated at room temperature for 45 minutes, at which point a volume of 100 × 10^−3^ m phosphate buffer (supplemented with 1 × 10^−3^ m EDTA, pH 8.0) equal to 3 volumes of the RNA plus polymer mixture was added and incubated for 15 min at room temperature to raise the pH to neutrality before dilution to final desired concentration.

### Preparation of Polymer 2‐RNA Conjugates

Polymer **2** was dissolved to 50 mg mL^−1^ in ethanol and diluted to 5 mg mL^−1^ with HBG as described above. The annealed and concentrated oligonucleotide was diluted to 1 mg mL^−1^ in HBG and was mixed with the 5 mg mL^−1^ aqueous solution of polymer 2 in HBG at ratios appropriate to achieve the indicated polymer:RNA molar ratios and incubated for 1 h at room temperature to form the desired conjugates.

### Characterization of Polymer + RNA Nanoparticle (NP) Properties

For particle diameter and surface charge evaluation, polymer/3pRNA formulations were made as described above, but substituting 100 × 10^−3^ m HEPES (pH 8.0) for phosphate buffer and substituting 1 × 10^−3^ m EDTA for HBG as a diluent, as buffers containing phosphate, glucose, and/or chloride caused electrode corrosion. The hydrodynamic size and zeta potential of the resultant loaded NPs were measured by DLS and zeta potential analysis using a Malvern Zetasizer at concentrations of 250 µg mL^−1^ of total 3pRNA and polymer material for zeta potential measurements in a disposable folded capillary. Encapsulation of RNA in polymer/RNA complexes was measured by an intercalating fluorescent dye exclusion assay using the Quant‐it RiboGreen RNA assay kit in triplicate at indicated polymer:RNA molar ratios according to the manufacturer's instructions (Invitrogen). Conjugation efficiency of RNA in polymer‐RNA conjugates was measured by incubating 0.50 µg of conjugated RNA with 12.5 USP units of heparin sulfate for 15 min before subjecting the samples to an agarose gel electrophoresis mobility shift assay in triplicate (2% agarose gel prestained with SYBR‐Safe (Invitrogen)) and performing densitometry measurements using Fiji on the resultant gel images (Bio Rad Gel Doc EZ Imager) by subtracting background and linearly interpolating the intensity of the unconjugated band between zero and the intensity of an unconjugated control band containing no polymer.^[^ ^76^ ^]^ Reductive disulfide bond cleavage was demonstrated with the same electrophoretic mobility shift assay, but additionally including 10 × 10^−3^ m TCEP during the heparin incubation step and extending the incubation to 30 min.

### RNase A Protection Assay

The ribonuclease protection assay was performed by incubating 1 µg of conjugated or complexed RNA with 5 units of RNase A (Thermo Scientific) for 1 h at 37 °C in optiMEM. These RNase reactions were terminated by supplementing each reaction mixture with 50 USP units of heparin sulfate, a final concentration of 10 × 10^−3^ m TECP, and a final concentration of 0.1% Triton‐X 100 for 1 h at 75 °C before quantifying remaining RNA by the electrophoretic mobility shift and densitometry assays described above, linearly interpolating band intensity between two control lanes of 1 µg free RNA with or without 5 units of RNase A.

### Cell Culture

A549 lung carcinoma cells, the IRF‐reporting A549‐Dual cell line (Invivogen), and engineered Gal8‐MDA‐MB‐231 cells.^[^ ^51^ ^]^ were cultured in DMEM supplemented with 4.5 g L^−1^ D‐glucose, 10% HI‐FBS, 2 × 10^−3^ m l‐glutamine, 100 U mL^−1^ penicillin, 100 µg mL^−1^ each of streptomycin, and 25 × 10^−3^ m HEPES at 37° C in a humidified atmosphere of 5% CO2. A549‐Duals were also supplemented with 100 µg mL^−1^ Normocin (Invivogen). Generally, 10^4^ cells were plated into each well of 96‐well cell culture microplates (Grenier) and allowed to adhere overnight before being transfected in quadruplicate or sextuplicate with indicated doses and polymers. Dose–response sweeps with 3pRNA NPs consisted of serial 2‐fold dilutions of polymer or polymer/RNA formulations described above with a typical maximum 3pRNA dose of 100–500 × 10^−9^ m after dilution in optiMEM. Treated cells were allowed to incubate for 24 h before measurements. In the case of serum challenge (Figure 6), formulations were diluted 1:1 with either FBS or 1X PBS and incubated at 37 °C for 4 h before dilution to the desired final concentration in optiMEM.

### Gal8 Recruitment Assays

Gal8 recruitment assay was performed as previously described with modifications.^[^ ^51^ ^]^ Treatments included either self‐assembled polymer NPs or polymers loaded with 5′ Alexa Fluor 647 RNA (Integrated DNA Technologies). After incubation for 18 h following treatment, the media was aspirated and replaced with FluoroBrite DMEM supplemented with 10% HI‐FBS, 2 × 10^−3^ m l‐glutamine, 100 U mL^−1^ penicillin, 100 µg mL^−1^ of streptomycin, 25 × 10^−3^ m HEPES, and 4 µM Hoechst 33342 (ThermoFisher Scientific). Cells were imaged on an ImageXpress Micro XLS Widefield High‐Content Analysis System outfitted with a 20× objective lens, courtesy of the Vanderbilt High Throughput Screening Core. Four replicate images were taken per well and each were analyzed using the Transfluor Application module of the MetaXpress software package to identify and quantify the fluorescent puncta in each image, which were subsequently normalized to the number of nuclei per image. Replicate images were also analyzed using the Multi Wavelength Cell Scoring module of the MetaXpress software to identify and quantify the cytosolic AF647 signal associated with each cell. These values were then used to establish fluorescent puncta per cell and mean cell‐average integrated intensity values, respectively. Each image was treated as an independent replicate, and a threshold cytosolic AF647 fluorescence intensity was established to create AF647^+^ and AF647^−^ categories for each cell.

### In Vitro Evaluation of Polymer and Polymer/3pRNA Formulations

Luminescent reporter assays using A549‐Duals were performed 24 h after treatment with indicated polymer/3pRNA formulation at indicated doses using QUANTI‐Luc luciferase detection reagent (Invivogen) according to the manufacturer's instructions. Cell viability assays were performed using CellTiter‐Glo reagent (Promega) according to the manufacturer's instructions. Measurements were taken using a Synergy H1 microplate reader (BioTek, Winooski, VT). All measurements within each experiment were taken with identical gain to normalize signal between treatment groups. All relative measurements were normalized by linear interpolation between the maximum mean value and untreated control. Curve fitting was performed in GraphPad Prism 9. Cell viability curves were fitted using the 4‐parameter variable slope logistic curve fitting as [Inhibitor] versus response. Dose–response curves were fitted using either the 4‐parameter variable slope logistic curve fitting as [Agonist] versus response for sigmoid‐type responses (including all tested pol:3pRNA ratios formulated with polymer **1** and pol:3pRNA ≤ 10 with polymer **3**) or the bell‐shaped curve fitting for bell curve‐type responses (including all tested pol:3pRNA ratios formulated with polymer **2** and pol:3pRNA = 12.5, 15 with polymer **3**).

### Flow Cytometry

2.5 × 10^5^ A549 cells were plated into 12‐well cell culture plates (Grenier) and allowed to adhere overnight before the following treatment schedule. At indicated timepoints, three wells were each aspirated of media and washed with 1x PBS before addition of 5′Cy5 RNA formulated with indicated polymer freshly diluted to 20 × 10^−9^ m RNA with complete cell culture medium (1 mL per well). Each well was treated with an independent formulation of polymer and 5′Cy5 RNA. Following treatment, cells were suspended in 50 µL media containing Fc block for 15 minutes at 4 °C. 50 µL of media containing fixable viability dye (APC‐Cy7; ThermoFisher) was added for 30 min at 4 °C. Cells were centrifuged at 380 × *g* for 5 min and rinsed in flow buffer (2% FBS in PBS + 0.5% Sodium Azide) and centrifuged again at the same conditions. Cells were then fixed in 2% paraformaldehyde for 10 min at room temperature in the dark. Cells were centrifuged at 650 × *g* for 5 min, rinsed twice in flow buffer, then centrifuged at 650 × *g* for 5 min. Samples were then measured using an Amnis CellStream (Cytek Biosciences) and analyzed using FlowJo (BD Biosciences).

### Animals

6‐ to 8‐week‐old Female C57BL/6 mice were obtained from the Jackson Laboratory (Bar Harbor, ME). All animals were housed and treated in compliance with the rules and regulations set forth by the Vanderbilt University Institutional Animal Care and Use Committee.

### Preparation of (3p)RNA NPs for In Vivo Injection

Thiolated 5′OH‐ or 5′3pRNA was concentrated to 10 mg mL^−1^ in CE buffer before addition to polymer **3** (in the case of 5′OH‐RNA) or to polymer **1**, **3**, or **4** (in the case of 3pRNA) prediluted to 25 mg mL^−1^ in CE with rapid mixing. The polymer to RNA molar ratio was 10 in all cases. Polymer/RNA formulations were allowed to incubate at room temperature for 1 hour and a reference aliquot was taken and stored at 4 °C before dialysis in small‐volume dialysis cassettes (Slide‐A‐Lyzer, 3000 MWCO, ThermoFisher Scientific), once against a 1500× volumetric excess of sterile‐filtered 5% d‐glucose (w/v) supplemented with 10 × 10^−3^ m HEPES and adjusted to pH 7.4 with iso‐osmolar 150 × 10^−3^ m KOH for 4 h, and once more against a 1000× volumetric excess of unbuffered sterile‐filtered 5% d‐glucose overnight. The concentration of 3pRNA was determined by quantification using RiboGreen RNA quantification reagent using the supplied TE buffer as a diluent for both the reference aliquot (as a standard) and sample, and TE + 2% Triton‐X 100 as the reagent diluent.^[^ ^77^ ^]^

### In Vivo Activity of 3pRNA + Polymer NPs

For the experiment comparing 5′OH‐ and 5′3pRNA NPs formulated with polymer **3** (Figure S12, Supporting Information), the NP solutions were diluted to 150 µg 3pRNA mL^−1^ in sterile 5% d‐glucose and administered to preweighed 8‐week‐old female C57BL/6 mice by retro‐orbital injection at 0.75 mg kg^−1^ 3pRNA. For the experiment examining 3pRNA formulated with polymers **1**, **3**, and **4** (Figure 7), the NP solutions were diluted to 240 µg 3pRNA mL^−1^ in sterile 5% d‐glucose and administered to preweighed 8‐week‐old female C57BL/6 mice by retro‐orbital injection at 1.2 mg kg^−1^ 3pRNA. Mice were treated with 10 µL of injection volume per 2 g body weight in both cases. 4 h post injection, mice were euthanized, and whole blood was collected by cardiac puncture into K_2_EDTA‐coated tubes (BD Biosciences), and the plasma was separated and collected by centrifugation at 2000 × *g* for 15 min at 4 °C. In the experiment comparing NPs with 5′OH‐ or 5′3pRNA NPs (Figure S12, Supporting Information), blood plasma levels of IFN‐α and IFN‐β were measured using LumiKine Xpress mIFN‐α 2.0 and LumiKine Xpress mIFN‐β 2.0 ELISA kits (Invivogen) using 2% bovine serum albumin (Research Products International) + 0.01% Tween‐20 (v/v, Sigma Aldritch) as a reagent diluent but otherwise according to the manufacturer's instructions. In the experiment comparing polymers **1**, **3**, and **4** (Figure 7), collected plasma was evaluated for concentrations of indicated cytokines using the LEGENDplex Mouse Anti‐Virus Response Panel (BioLegend) according to the manufacturer's instructions (v‐bottom plate method), and the data were collected using the flow cytometer listed above. Cytokine concentrations were interpolated from standard curves generated using asymmetric sigmoidal 5‐paramater logistic curve fits in GraphPad Prism 9. Outliers were identified using the ROUT method (*Q* = 1%) and pruned from all datasets before any further analysis. GM‐CSF measurements are not shown, as there were no notable elevations above zero observed in any test group.

### Statistics

Statistical comparisons were performed using GraphPad Prism 9 software according to the methods indicated in the figure captions for each respective experiment with *P* < 0.05 set as the threshold for statistical significance.

## Conflict of Interest

C.R.P. and J.T.W. are authors on a pending patent application encompassing some of this work (U.S. Patent Application No. 63/579,876).

## Supporting information

Supporting Information

## Data Availability

The data that support the findings of this study are available from the corresponding author upon reasonable request.
