# Supplementary material for: Covalent Polymer‐RNA Conjugates for Potent Activation of the RIG‐I Pathway
Source: Adv Healthc Mater. 2024 May 3;14(5):2303815. doi: 10.1002/adhm.202303815 (PMC11493851; doi:10.1002/adhm.202303815)
Supplement: Supplementary file 1 — Supporting Information [file ADHM-14-0-s001.pdf]

ADVANCED HEALTHCARE MATERIALS

Supporting Information

for *Adv. Healthcare Mater.*, DOI 10.1002/adhm.202303815

Covalent Polymer-RNA Conjugates for Potent Activation of the RIG-I Pathway

*Christian R. Palmer, Lucinda E. Pastora, Blaise R. Kimmel, Hayden M. Pagendarm, Alexander J. Kwiatkowski, Payton T. Stone, Karan Arora, Nora Francini, Olga Fedorova, Anna M. Pyle and John T. Wilson**

Supporting Information:

Covalent Polymer-RNA Conjugates for Potent Activation of the RIG-I Pathway

Christian R. Palmer[a], Lucinda E. Pastora[a], Blaise R. Kimmel[a], Hayden M. Pagendarm[b], Alexander J. Kwiatkowski[a], Payton T. Stone[a], Karan Arora[a], Nora Francini[b], Olga Fedorova[c,d], Anna M. Pyle[c,d,e], John T. Wilson[a,b,f,g,*]

[a]Department of Chemical and Biomolecular Engineering, Vanderbilt University, Nashville, TN 37235, USA
[b]Department of Biomedical Engineering, Vanderbilt University, Nashville, TN 37235, USA
[c]Department of Molecular, Cellular and Developmental Biology, Yale University, New Haven, CT
[d]Howard Hughes Medical Institute, Chevy Chase, MD
[e]Department of Chemistry, Yale University, New Haven, CT
[f]Department of Pathology, Microbiology, and Immunology, Vanderbilt University Medical Center, Nashville, TN 37232, USA
[g]Vanderbilt-Ingram Cancer Center, Vanderbilt University Medical Center, Nashville, TN 37232, USA
*Corresponding Author: john.t.wilson@vanderbilt.edu

Safety statement

No unexpected or unusually high safety hazards were encountered in this work.

Supplementary Figures

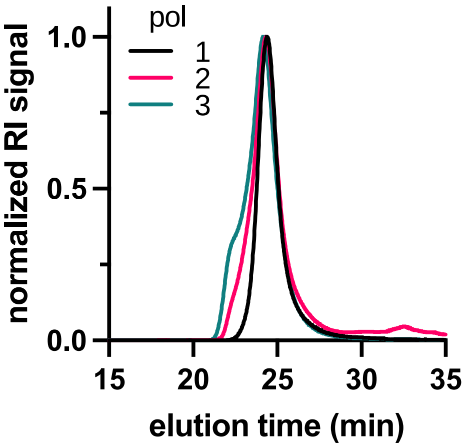

Figure S1. Gel permeation chromatograms of polymers 1-3. RI: refractive index.

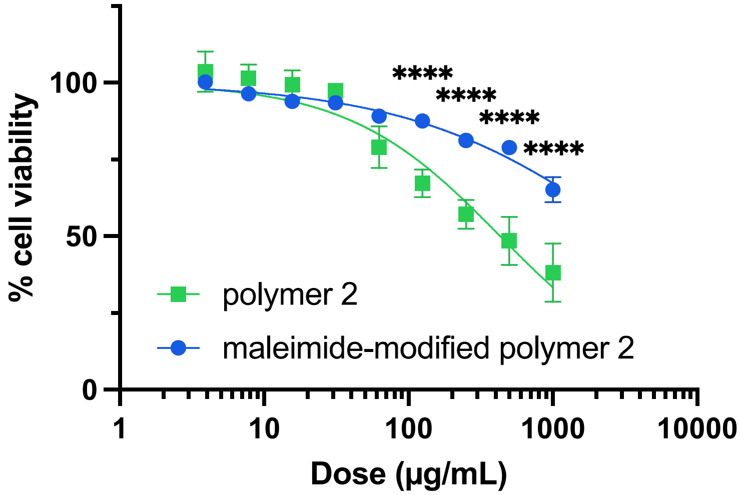

Figure S2: Viability of cell treated with polymer 2 and maleimide-modified polymer 2. Percent of viable A549 cells after treatment with indicated polymer at indicated doses. Comparisons made by 2-way ANOVA with Šidák's multiple comparisons test. n = 3 for each point. *** *P* < 0.0001. Data shown are mean +/- SD.

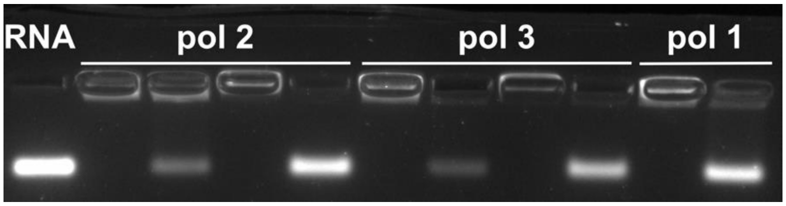

Figure S3. Electrophoretic mobility shift assay of 3pRNA formulated with polymers 1-3. Uncut original image.

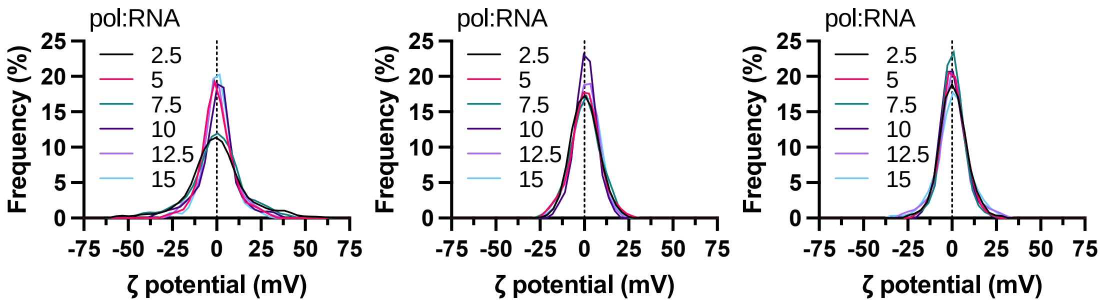

Figure S4. Representative zeta (ζ) potential histograms of polymer/3pRNA formulations. Representative histograms of ζ potential measurements of 3pRNA formulated with polymer **1** (left), polymer **2** (center), and polymer **3** (right) at indicated polymer:RNA molar ratios.

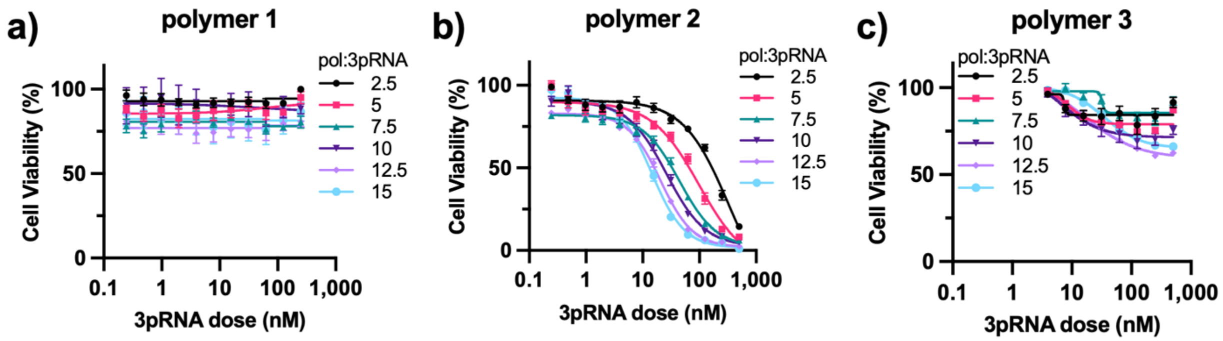

Figure S5. Viability of cells treated with polymer/3pRNA formulations. Representative percentage of viable cells after treatment of 3pRNA formulated with **a)** polymer **1**, **b)** polymer **2**, or **c)** polymer **3** at indicated doses and pol:3pRNA ratios. All data shown are mean +/- SD.

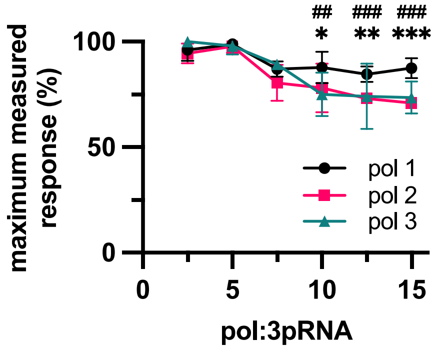

Figure S6. Maximum response of reporter cells treated with polymer/3pRNA formulations. Maximum measured responses of A549-Dual reporter cells treated with 3pRNA formulated with indicated polymer at indicated pol:3pRNA ratios across all tested doses. Asterisks (*) indicated comparisons between polymer **1** and polymer **2**. Hashes (#) indicate comparisons between polymer **1** and polymer **3**. * $P < 0.05$. **, ## $P < 0.01$. ***, ### $P < 0.001$. Comparisons shown are by 2-way ANOVA with Tukey's multiple comparisons test with each pol:3pRNA ratio constituting one family. n = 2 for polymer **3**:3pRNA ratio = 2.5. n = 3 for all other points. All data shown are mean +/- SD.

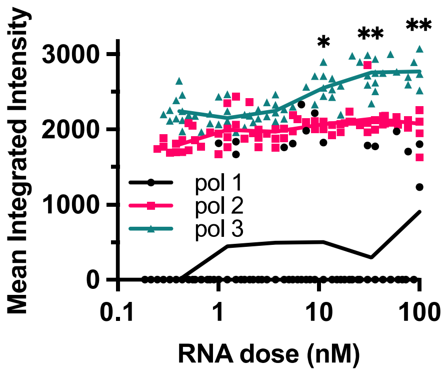

Figure S7. Mean cell-average integrated intensity of cells treated with 5'AF647-RNA/polymer formulations. Cell-averaged integrated intensities of each replicate image are shown as individual data points. Comparisons are made by two-way ANOVA with Dunnett's multiple comparisons test. Comparisons are made between each polymer with each dose constituting one family. Asterisks (*) indicate comparisons between polymers **2** and **3**. * $P < 0.05$. ** $P < 0.01$.

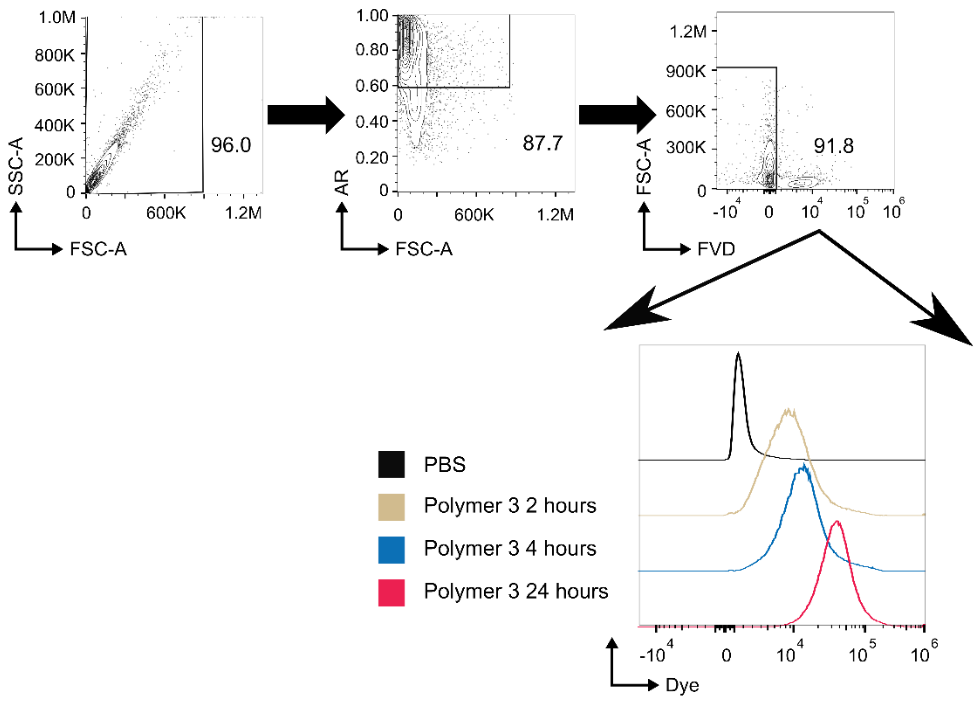

Figure S8. Flow cytometric analysis of A549 Cells. Representative flow cytometry showing gating schemes related to main text **Figure 4h-j** for analysis of the frequency of NP uptake. SSC: side scatter. FSC: forward scatter. AR: Aspect Ratio.

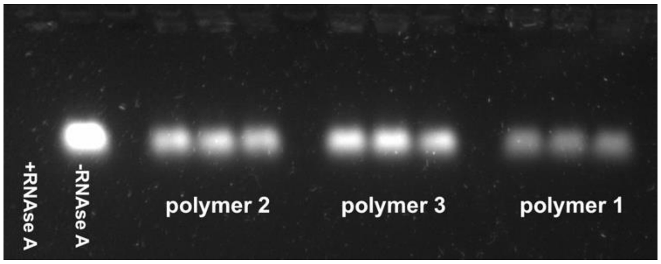

Figure S9. Electrophoretic mobility shift assay following RNAse A challenge of 3pRNA formulated with polymers 1-3. Uncut original image.

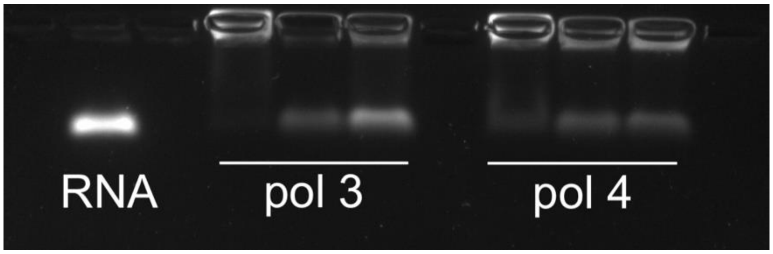

Figure S10. Electrophoretic mobility shift assay of polymers 3 and 4. Uncut original image.

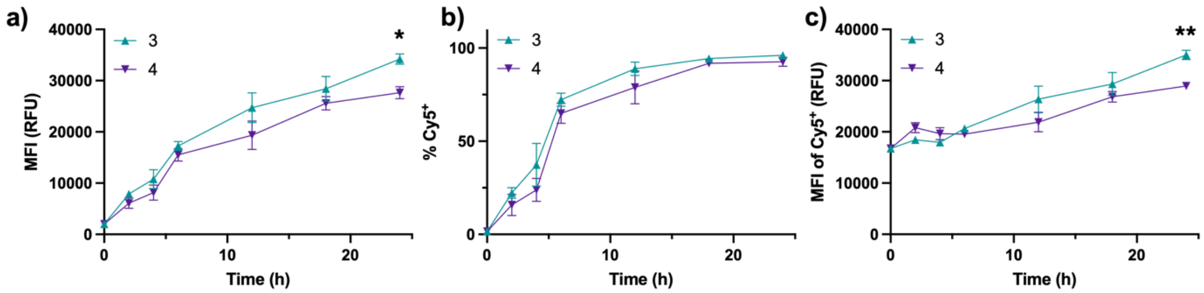

Figure S11. Cellular uptake of Cy5 RNA conjugated to polymers 3 and 4. a) Median fluorescence intensity (MFI), b) percentage of cells testing positive for Cy5 (% Cy5^{+}), and c) MFI of Cy5^{+} cells after treatment with Cy5 RNA conjugated to indicated polymer for indicated duration. Comparisons made by two-way ANOVA with Šidák's multiple comparisons test. * $P < 0.05$. ** $P < 0.01$. n = 3 for each point.

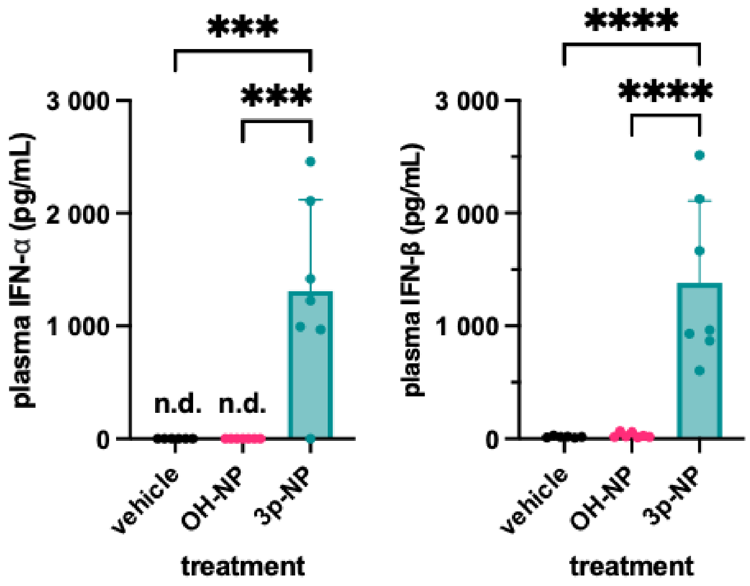

Figure S12. In vivo activity of polymer 3–3pRNA conjugates. Plasma Interferon-α (IFN-α, left) and Interferon-β (IFN-β, right) concentrations of following intravenous administration of indicated treatment (0.75 mg 3pRNA kg^{-1} body mass) as measured by enzyme-linked immunosorbent assay. vehicle: 5% dextrose (w/w). n = 5 (veh), n = 5 (OH-NP), n = 7 (3p-NP). Comparisons shown are by ordinary one-way ANOVA with Tukey's multiple comparisons test. Data shown with mean +/- SD. *** $P < 0.001$. **** $P < 0.0001$.

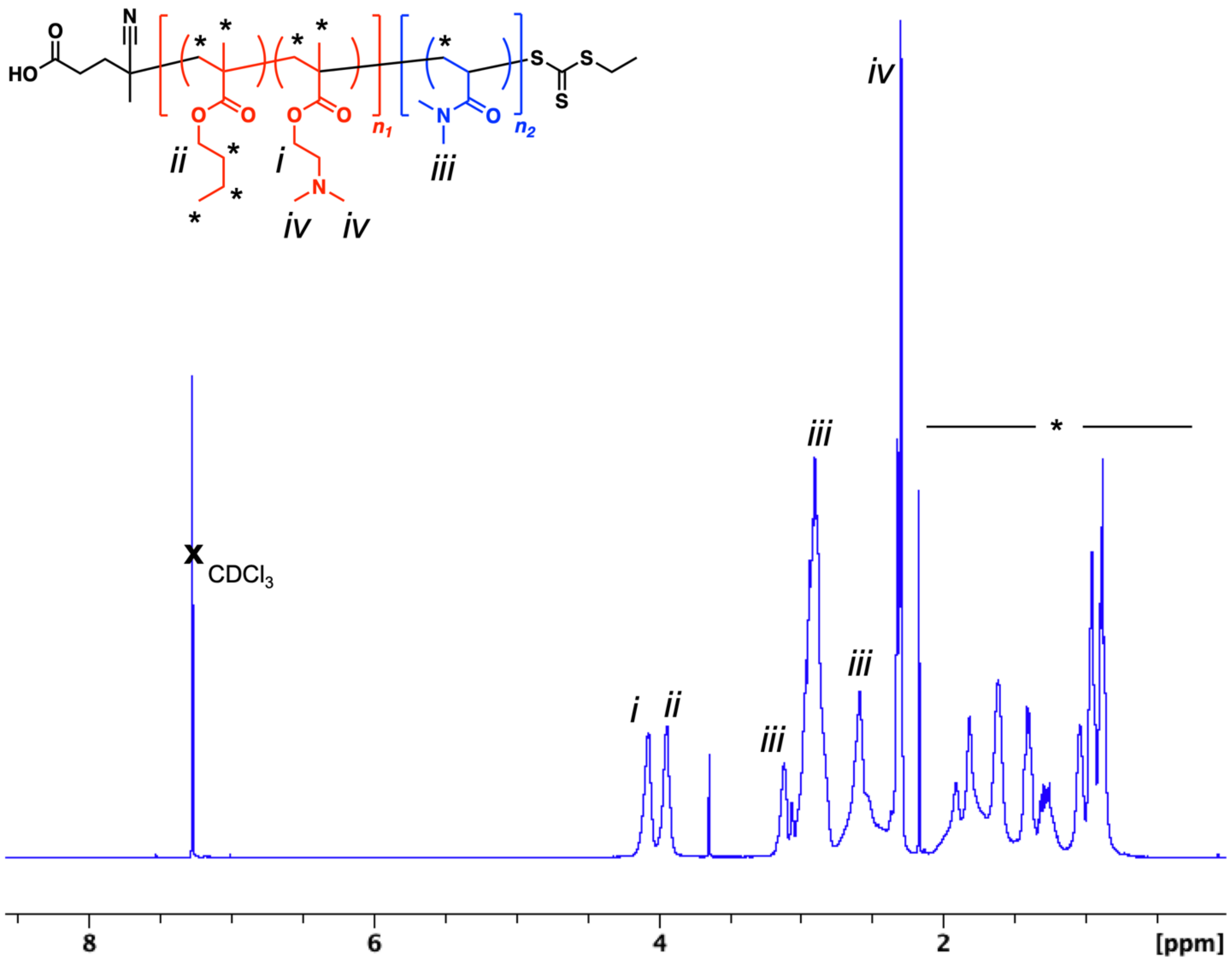

Figure S13. ¹H-NMR spectrum of polymer 1. Chemical shifts are reported as parts per million (ppm) versus tetramethyl silane (TMS). Spectrum acquired in Chloroform-d ($CDCl_3$).

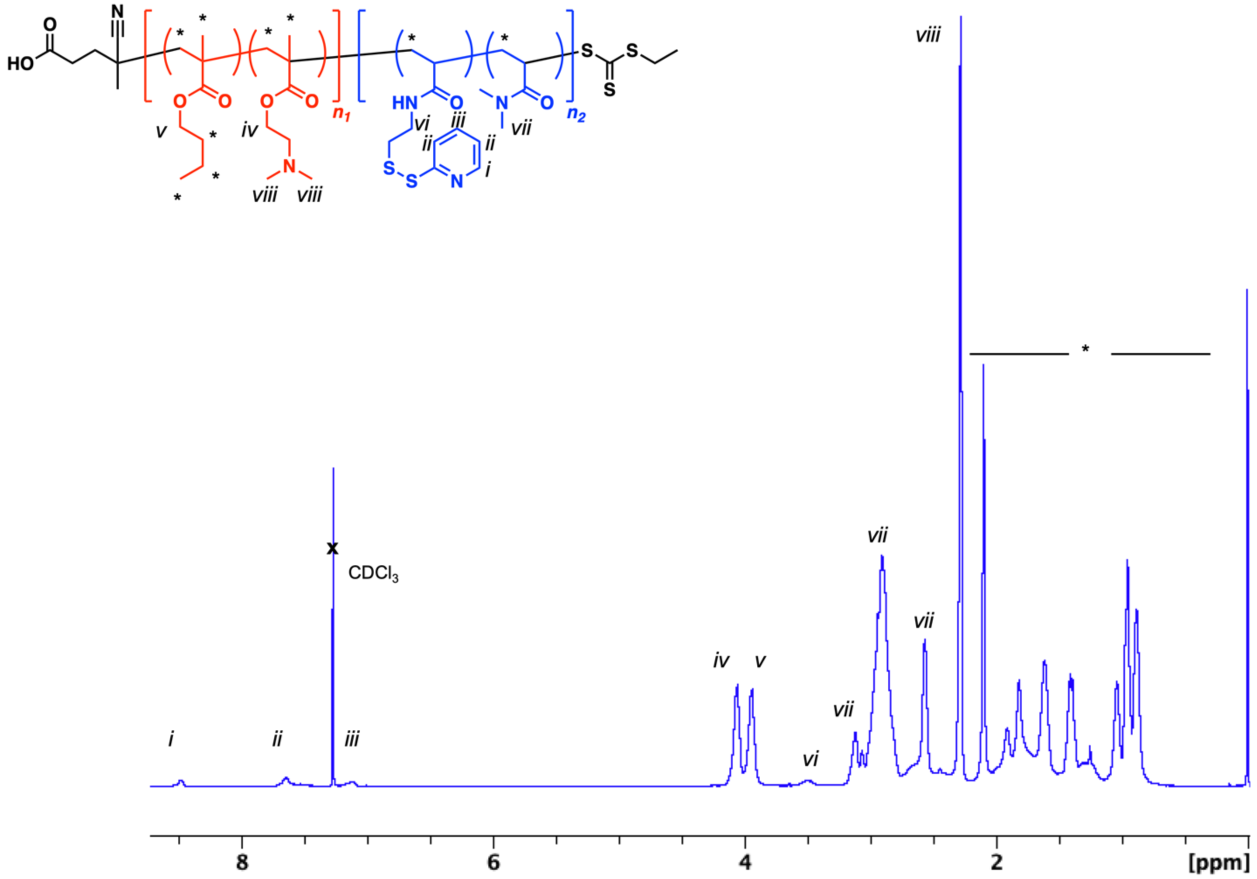

Figure S14. ^{1}H-NMR spectrum of polymer 2. Chemical shifts are reported as parts per million (ppm) versus tetramethyl silane (TMS). Spectrum acquired in Chloroform-d ($CDCl_3$).

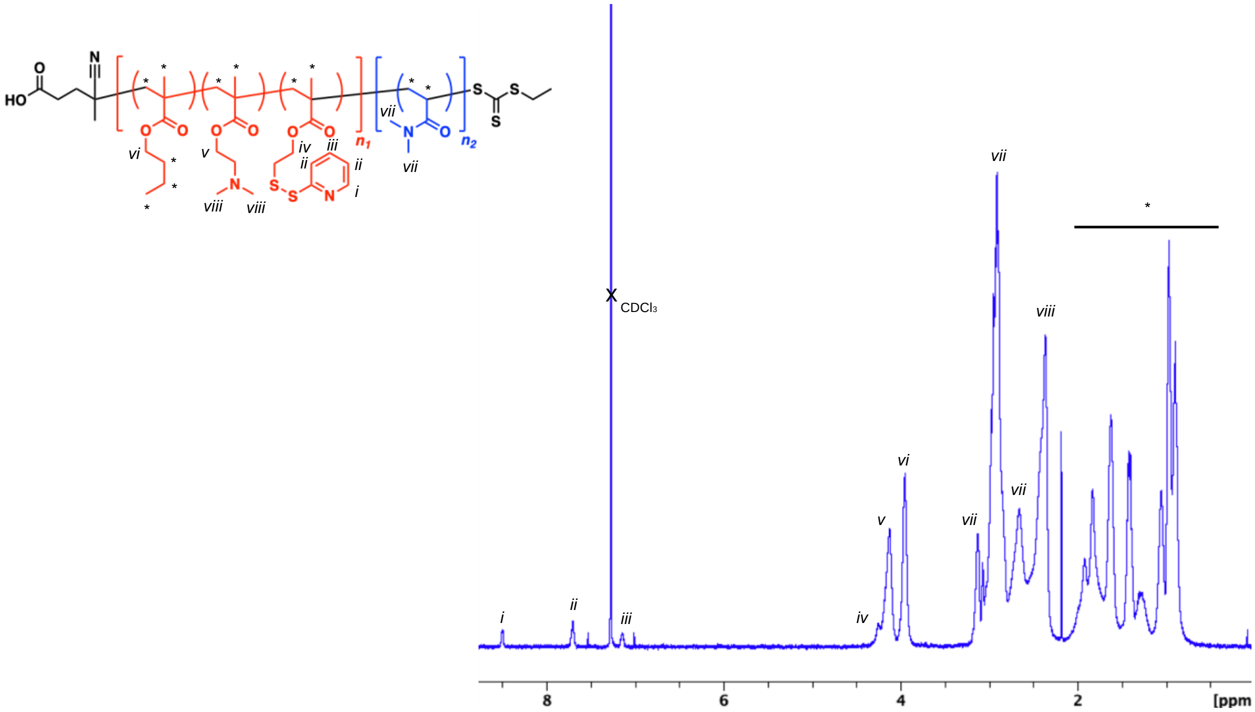

Figure S15. ¹H-NMR Spectrum of polymer 3. Chemical shifts are reported as parts per million (ppm) versus tetramethyl silane (TMS). Spectrum acquired in Chloroform-*d* ($CDCl_3$).

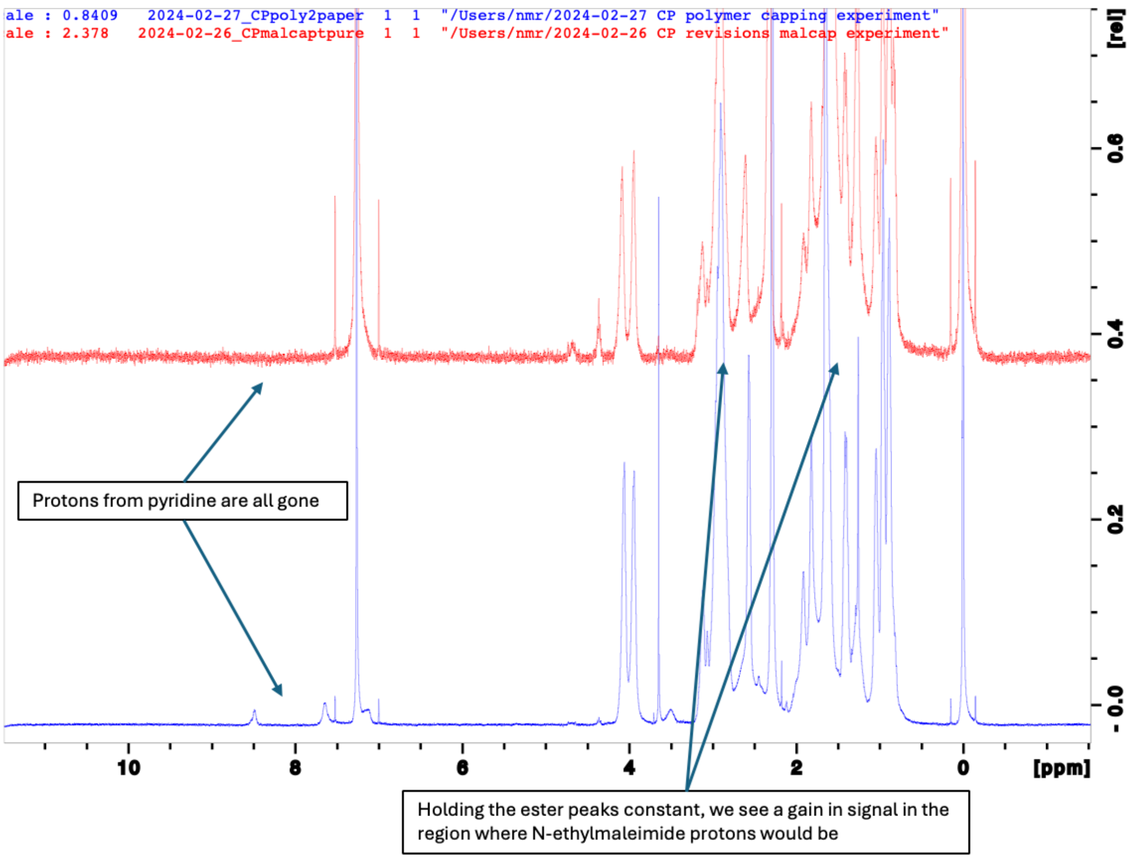

Figure S16. ^{1}H-NMR Spectrum of maleimide-modified polymer 2. Chemical shifts are reported as parts per million (ppm) versus tetramethyl silane (TMS). Spectrum acquired in Chloroform-*d* ($CDCl_3$).

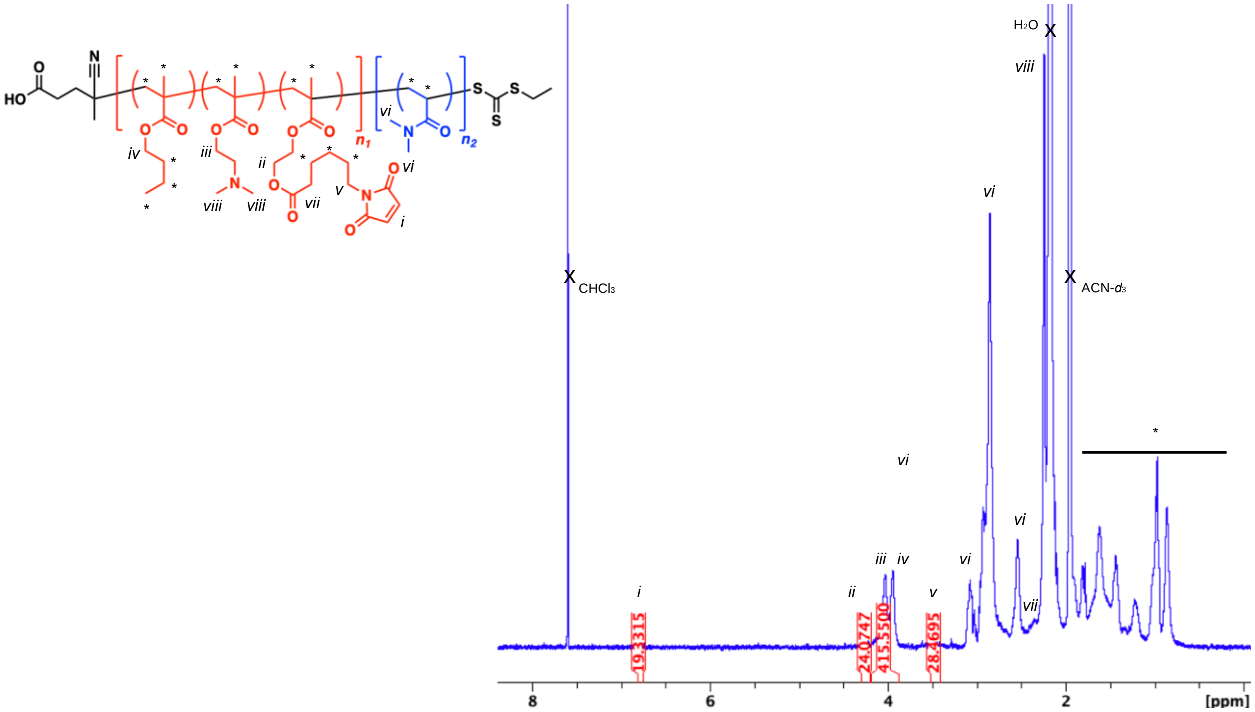

Figure S17. ^{1}H-NMR Spectrum of polymer 4. Chemical shifts are reported as parts per million (ppm) versus tetramethyl silane (TMS). Spectrum acquired in acetonitrile-d_3 (ACN-d_3).

Supplementary Tables

polymer	DP_1	DP_2	conjugation sites	$M_{n,calc.}$ (kg mol^{-1})	$M_{n,GPC}$ (kg mol^{-1})
1	225	188	-	52.5	53.6
2	235	189	10.4	55.4	57.0
3	210	190	9.86	51.6	65.7
4	208	186	12.0	52.0	-

Table S1. Summary of polymer properties. DP_1: core-forming block degree of polymerization (DP). DP_2: corona-forming block DP. $M_{n,calc.}$: estimated number-average MW (M_n) as calculated by conversion ^{1}H NMR. $M_{n,GPC}$: estimated M_n from gel-permeation chromatography (GPC) measurements as compared to poly(methyl methacrylate) standards. Conjugation sites denotes count of PDS groups, except in the case of polymer **4**, where it denotes count of maleimide groups.

polymer	M_0: CTA	CTA	initiator	ºC	wt% toluene	mol% DMAEMA	mol% BMA	mol% PDSEMA	mol% HEMA	mol% DMA	mol% PDSEAm	% conversion
mCTA1	250	ECT	AIBN	75	50	50	50	0	0	0	0	89.8
mCTA2	250	ECT	AIBN	75	50	50	50	0	0	0	0	94.2
mCTA3	250	ECT	AIBN	75	50	47.5	47.5	5.0	0	0	0	84.1
mCTA4	250	ECT	AIBN	75	50	47.5	47.5	0	5.0	0	0	83.2
1	200	mCTA 1	AIBN	75	60	0	0	0	0	100	0	94.1
2	232	mCTA 2	V-70	40	60	0	0	0	0	94.2	5.8	82.0
3	200	mCTA 3	V-70	40	60	0	0	0	0	100	0	94.9
4-OH	200	mCTA 4	V-70	40	60	0	0	0	0	100	0	93.0

Table S2. Summary of polymerization conditions. M_0: initial monomer. CTA: chain transfer agent. DMAEMA: 2-(dimethylamino)ethyl methacrylate. BMA: Butyl methacrylate. PDSEMA: PDS-ethyl methacrylate. HEMA: 2-hydroxyethyl methacrylate. DMA: N,N-dimethylacrylamide. PDSEAm: PDS-ethyl acrylamide.
